# Silicon oxycarbide glass-graphene composite paper electrode for long-cycle lithium-ion batteries

**DOI:** 10.1038/ncomms10998

**Published:** 2016-03-30

**Authors:** Lamuel David, Romil Bhandavat, Uriel Barrera, Gurpreet Singh

**Affiliations:** 1 Mechanical and Nuclear Engineering Department, Kansas State University, 3002 Rathbone Hall, Kansas, Manhattan, Kansas 66506, USA

## Abstract

Silicon and graphene are promising anode materials for lithium-ion batteries because of their high theoretical capacity; however, low volumetric energy density, poor efficiency and instability in high loading electrodes limit their practical application. Here we report a large area (approximately 15 cm × 2.5 cm) self-standing anode material consisting of molecular precursor-derived silicon oxycarbide glass particles embedded in a chemically-modified reduced graphene oxide matrix. The porous reduced graphene oxide matrix serves as an effective electron conductor and current collector with a stable mechanical structure, and the amorphous silicon oxycarbide particles cycle lithium-ions with high Coulombic efficiency. The paper electrode (mass loading of 2 mg cm^−2^) delivers a charge capacity of ∼588 mAh g^−1^_electrode_ (∼393 mAh cm^−3^_electrode_) at 1,020th cycle and shows no evidence of mechanical failure. Elimination of inactive ingredients such as metal current collector and polymeric binder reduces the total electrode weight and may provide the means to produce efficient lightweight batteries.

## Abstract (web summary)

Most high-loading silicon-based anodes for lithium-ion batteries suffer from low efficiency and volumetric capacity. Here, the authors show that a paper-like electrode of silicon oxycarbide glass and graphene at mass loading of >2 mg cm^−2^ can efficiently deliver high energy density for over 1,000 cycles.

## Graphical abstract

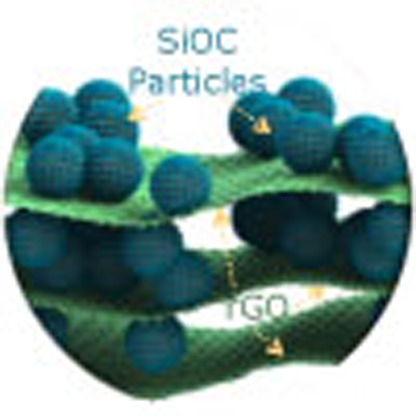

Concentrated efforts are currently employed to discover a practical replacement for traditional Li-ion battery electrodes that is, graphite anode and LiCoO_2_ cathode with materials that continuously deliver high power and energy densities at high cycling efficiencies without damage^1^,^2^,^3^,^4^,^5^. Alloying reaction electrodes such as silicon that can deliver as much as 5–10 times higher discharge capacity than traditional graphite, are at the forefront of this research. High capacity electrodes, however, are prone to enormous volume changes (∼300%) that generally lead to structural collapse and capacity fading during successive lithiation/delithiation^6^,^7^,^8^,^9^,^10^,^11^,^12^. Recent work has shown that decreasing particle size or electrode nanostructuring allows the electrode to withstand high volumetric strains associated with repeated Li alloying and de-alloying. Pomegranate-inspired carbon-coated Si nanoparticles, yoke shell-structured SiC nanocomposites and Si/C core/shell composites (prepared at low mass loading) have proven to survive several hundred cycles without damage^9^,^10^,^11^,^12^,^13^. Yet, electrode nanostructuring has lead to new fundamental challenges such as low volumetric capacity (low tap density), increased electrical resistance between the nanoparticles, increased manufacturing costs and lower Coulombic efficiency due to side reactions with the electrolyte. These challenges have not been fully addressed. What's more, a particle-based electrode's long-term cyclability hinges on the inter-particle electrical connection and particle adhesion to the metallic substrate, which decreases rapidly with increasing charge/discharge cycles, particularly for thick high-loading electrodes^9^.

In this context, the graphene-based multicomponent composite anodes are an attractive alternative to traditional (binder and carbon-black) designs, chiefly because of graphene's superior electronic conductivity, mechanical strength and ability to be interfaced with Li active redox components, such as particles of Si, Ge, and transition metals sulfides/oxides resulting in electrodes that are intrinsically conducting and promote faster ion diffusion^14^,^15^,^16^,^17^,^18^,^19^,^20^,^21^,^22^,^23^,^24^,^25^,^26^,^27^,^28^,^29^,^30^,^31^,^32^,^33^,^34^,^35^,^36^,^37^,^38^. Additional advantages include weight savings of up to 10% of the total battery weight^7^, if the electrode is prepared in the freestanding form, improved corrosion resistance (elimination of metal foil), and enhanced flexibility, particularly for bendable, implantable, and roll-up electronics.

In spite of these advantages, graphene-paper electrodes do not offer an absolute solution because of the following associative disadvantages: (a) potential limiting of overall battery capacity due to insufficient active mass (thickness generally limited to submicrometers), (b) expensive techniques required for synthesis of Li-redox components and (c) more important, paper anodes generally show very high first cycle loss (50–60%), low cycling efficiency (95–98%) and poor capacity retention at high current densities (damage at high C-rates)^23^,^24^,^25^,^26^,^27^,^28^,^29^,^30^,^31^,^32^,^33^,^34^,^35^,^36^,^37^,^38^,^39^, making graphene-paper electrodes somewhat impractical for use in an Li-ion battery full-cell. Here again, very few studies have been performed to investigate the mechanical and fracture properties of composite paper-based electrodes.

Continued search for better anodes has brought attention to unique, rarely studied molecular precursor-derived Si-based glass-ceramics (such as silicon oxycarbide or SiOC and silicon carbonitride or SiCN) materials^40^,^41^,^42^,^43^,^44^,^45^,^46^,^47^,^48^,^49^,^50^. SiOC is a high-temperature glass-ceramic with an open polymer-like network structure consisting of two interpenetrating amorphous phases of SiOC (Si bonded to O and C) and disordered carbon^42^. Its low weight density (∼2.1 g cm^−3^) and open structure enables high charge and discharge rates with a gravimetric capacity more than twice that of commercial graphite electrode. More important, major portion of the electrochemical capacity in SiOC is due to reversible Li-adsorption in the disordered carbon phase and not the conventional alloying reaction with Si, ensuing relatively lower volumetric changes^43^,^44^. Regrettably, the glass-ceramics that show high lithiation capacity are poor conductors of electronic/ionic current and consequently the electrode preparation involves incorporation of conducting agents and binders in order to hold the particles on a metal current collector, a method known as screen printing^45^,^46^,^47^. Such foil-based electrodes carry the dead weight of conducting agents, polymeric binders, and the metal foil that do not contribute towards the battery capacity.

As an attractive solution to screen printed electrodes, we present our results related to fabrication of a well-organized, interleaved, freestanding, large-area composite anode consisting of SiOC particles supported by crumpled reduced graphene oxide matrix. The electrode delivers higher volumetric capacity than the recently reported pomegranate Si/carbon nanotube (310 mAh cm^−3^) paper-electrode^9^. Large micrometer size reduced graphene oxide (rGO) sheets serve as host material to SiOC particles, providing the necessary electronic path and consistent cycling performance at high current densities along with high structural stability. Because of their unique nanodomain amorphous structure, SiOC particles offer required chemical and thermodynamic stability and high Li intercalation capacity for the electrode. As a result the electrode (at least 2 mg cm^−2^ weight loading) has first cycle charge capacity of 702 mAh g^−1^_electrode_ (total weight of electrode considered) and ∼470 mAh cm^−3^_electrode_ (total volume of electrode considered) at 100 mA g^−1^_electrode_ and stable charge capacity of 543 mAh g^−1^_electrode_ (∼363 mAh cm^−3^_electrode_) at charge current density of 2,400 mA g^−1^_electrode_. The capacity is ∼200 mAh g^−1^_electrode_ when cycled at ∼-15 °C. Further, the composite electrode has exceptionally high strain-to-failure (exceeds 2%) as measured in a uniaxial tensile test and the mode of failure differ significantly from pristine rGO papers.

## Results

### Material synthesis and electrode fabrication

**Figure 1:**
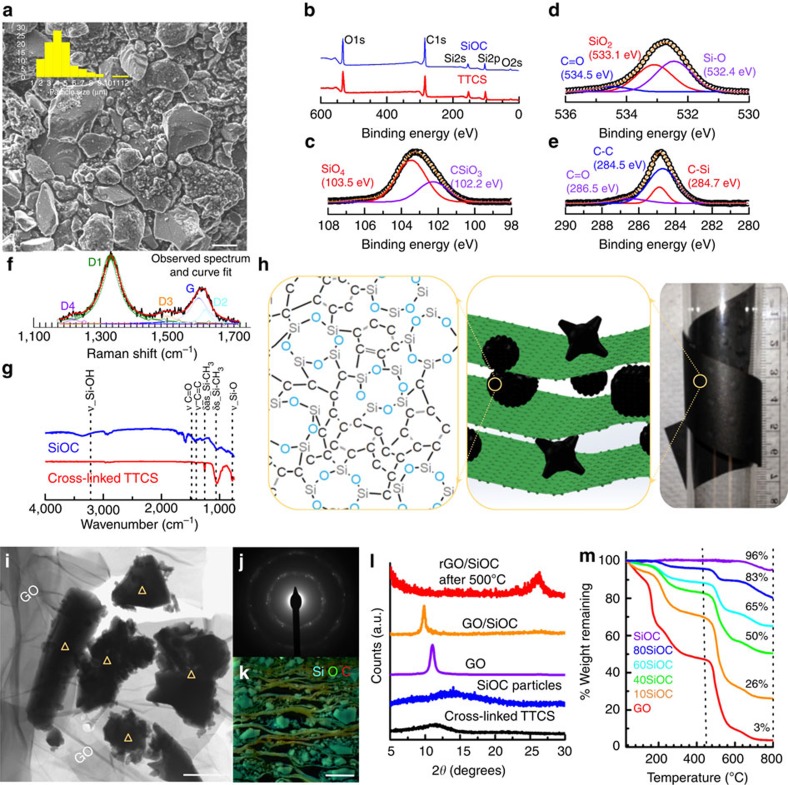
Characterization of SiOC ceramic and SiOC/graphene composite papers. (**a**) SEM image of SiOC particles after pyrolysis of the polymeric TTCS particles. Sharp glass-like particles decorated with sub-micron size particles were observed. Scale bar is 5 μm. (**b**) XPS survey scans for cross-linked TTCS and pyrolyzed SiOC. High resolution XPS spectrum of pyrolyzed SiOC particles in the (**c**) Si 2*p* region, (**d**) O 1*s* region and (**e**) C 1*s* region were consistent with the polymer-derived SiOC nanodomain model. Deconvoluted peaks indicate the various bonds between Si, C and O atoms that are distinct to pyrolyzed SiOC. (**f**) Raman spectrum of SiOC showed peaks that are characteristic of graphite-like carbon (D1-peak: 1,350 cm^−1^ and G-peak: 1,590 cm^−1^). (**g**) Fourier Transform Infrared Spectroscopy spectra of SiOC and cross-linked TTCS (*ν*: stretching vibration mode and *δ*: bending vibration mode). (**h**) Digital camera picture and schematic illustration of proposed hybrid structure of the freestanding paper along with the atomic structure of pyrolyzed SiOC particle. (**i**) TEM image of SiOC/GO composite material. Large GO flakes covering SiOC particles (Δ) were observed. Scale bar is 500 nm. (**j**) Corresponding TEM selected area electron diffraction pattern showed multiple spot pattern due to polycrystallinity of restacked GO sheets with faint ring pattern attributed to amorphous SiOC material. (**k**) FIB cross-sectional EDX elemental map of 60SiOC paper in which Si, C and O are indicated by blue, red and green, respectively. The scale bar is 5 μm. Additional TEM and SEM images are presented in Supplementary Figs. 6–8. (**l**) XRD of cross-linked TTCS, SiOC particles, GO and composite papers before and after thermal reduction (annealing). Complete reduction of GO to rGO is illustrated in the plot. (**m**) TGA curves of GO paper and unannealed composite paper measured from 30 to 800 °C (10 °C min^−1^) in flowing air (20 ml min^−1^). The weight percentage of SiOC in the unannealed composite is as indicated in the figure.

Polymer-derived SiOC ceramic particles were prepared by controlled thermolysis of 1,3,5,7-tetramethyl-1,3,5,7-tetravinylcyclotetrasiloxane (TTCS) polymeric precursor while graphene oxide (GO) was prepared by the modified Hummer's method^51^ (for details, see Methods section). The polymer-to-ceramic transformation was complete at 1,000 °C^41^. Detailed characterization of cross-linked polymer and resulting SiOC material is presented in Fig. 1a–g. SEM images of SiOC particles in Fig. 1a confirmed average particle size to be ∼4 μm (with s.d.=1.8 μm). X-ray photoelectron spectroscopy (XPS) showed O 1*s*, C 1*s*, Si 2*s*, Si 2*p* and O 2*s* peaks for both cross-linked and pyrolyzed SiOC ceramic (Fig. 1b). Close analysis of the deconvoluted silicon band (for Si 2*p* photoelectrons) in SiOC revealed the emergence of peaks at 103.5 and 102.2 eV, corresponding to SiO_4_ and CSiO_3_ phases, respectively (Fig. 1c). In addition, peaks at 534.5, 533.1 and 532.4 eV corresponding to C=O, SiO_2_ and Si–O phases, respectively, were observed in O 1*s* band (Fig. 1d), whereas the C 1*s* band (Fig. 1e) was fitted with 3 peaks at 286.5, 284.5 and 284.7 eV corresponding to C=O, C−C and C−Si phases, respectively. Surface elemental composition from XPS was measured to be C=62.55 at% (50.35 wt%), O=25.73 at% (27.57 wt%) and Si=11.72 at% (22.06 wt%). XPS composition after 80 min of depth profiling (with 5 keV Ar-ion) showed lower carbon and oxygen content of 50.78 at% (34.47 wt%) and 18.44 at% (16.66 wt%), respectively with Si at 30.78 at% (48.85 wt%) (see Supplementary Fig. 1). Bulk composition of SiOC particles was also determined from combustion and inert gas fusion techniques (see Supplementary Fig. 2a,b and Methods section for details). The composition was found to be C=51.24 at% (38.3 wt%), O=19.79 at% (19.7 wt%), H=5.09 at% (0.31 wt%) and Si=23.85 at% (41.68 wt%). The elemental composition obtained from various techniques is summarized in Supplementary Table 1. Raman spectroscopy of SiOC particles was performed to further confirm the existence of the free or excess carbon domains. As shown in Fig. 1f, five peaks could be fitted into the spectrum: D1 or D-band (∼1,330 cm^−1^), D2 (∼1,615 cm^−1^), D3 (∼1,500 cm^−1^), D4 (∼1,220 cm^−1^) and the G-band (∼1,590 cm^−1^) 52. D1, D2 and D4 originate from disordered graphitic lattice (graphene layer edges, surface layers and polyenes and so on) while D3 is associated with amorphous carbon soot. G-band corresponds to the ideal graphitic lattice. In addition, two bumps centered at ∼2,640 (2*D overtone) and ∼2,915 cm^−1^ (D+G combination) were also observed (Supplementary Fig. 3). Similarly, Fourier Transform Infrared Spectroscopy (FTIR) analysis also confirmed transformation of TTCS polymer to ceramic SiOC (Fig. 1g)^41^. Based on spectroscopic evidence, the predicted chemical structure of the cross-linked polymer and resultant ceramic is presented in Supplementary Fig. 4, which is in agreement with previous work on polymer-derived SiOC^42^.

The composite papers were prepared following a vacuum filtration technique (see Materials section for details and schematic in Supplementary Fig. 5). Samples were labeled as rGO, 10SiOC, 40SiOC, 60SiOC and 80SiOC for rGO paper and GO with 10, 40, 60 and 80 wt% of SiOC in the paper, respectively. The digital camera image and schematic in Fig. 1h highlights the flexibility and structure of the composite paper, respectively.

Morphology of the composite and thermally reduced (annealed) freestanding papers was studied by electron and focused ion beam (FIB) microscopy. The transmission electron microscope (TEM) image (Fig. 1i) showed large micrometer-sized thin GO sheets along with random shape glass-like SiOC particles (also see Supplementary Fig. 6a–e). Large SiOC particles were seen to be covered with smaller nanometer size particles. The graphene platelets seem to ocassionaly fold and cover individual SiOC particles and other instances show GO being interlayered by SiOC. EDX elemental mapping performed in scanning-TEM mode (Supplementary Figs. 6a–e) confirmed the uniform distribution of Si, O, C in the particles with higher concentration of C observed near the edges possibly due to graphene platelets. For the selected area electron diffraction pattern in Fig. 1j, the multiple spot pattern is a result of polycrystallinity of restacked GO sheets and the faint ring pattern is attributed to amorphous SiOC material. The SEM images of the freestanding papers showed a sheet-like structure with a relatively smooth top surface for rGO paper^53^,^54^,^55^,^56^, which became increasingly rough and porous with higher loading of SiOC particles in the composite (Supplementary Fig. 7a–d). Cross-sectional SEM of the fractured samples revealed ordered stacks of rGO with SiOC particles interlayered between the sheets (Supplementary Fig. 7e–h). Several micrometer sized particles could be seen for 60SiOC specimen along with clumped nanometer sized particles. Nonetheless, mechanically fractured composite papers were largely uneven and showed signs of damage to the interface. To obtain a smooth and defect-free cross-section, the 60SiOC paper was sectioned by means of a FIB milling (see Methods section and Supplementary Fig. 8a for details regarding specimen preparation). The uniform distribution of SiOC particles and wrapping by large-area graphene platelets could be clearly observed in the electron beam (Supplementary Fig. 8b) and ion-beam images (Supplementary Fig. 8c). Elemental mapping by means of EDX (Fig. 1k and Supplementary Fig. 8d–f) further established the inter-layered morphology of the composite. Depending up on the SiOC content, the average thickness of the papers varied between ∼20 and 30 μm.

The reduction of GO (non-conducting) to rGO (conducting) was confirmed by use of X-ray diffraction (XRD). As shown in Fig. 1l, both GO and unannealed composite papers, had peaks at 11.05 and 9.8°, corresponding to interlayer spacing of 8 and 12 Å, respectively. Interlayer spacing was large compared with that of graphite (with major peak (002) at 26.53°, corresponding to 3.36 Å) because of oxygen functional groups present in GO and water molecules held between the layers. After thermal annealing at 500 °C for 2 h, the paper showed a broad peak at 2*θ*=26°, typical of reduced GO material^55^,^56^. The broad peak observed in the spectra suggests inhomogeneous spacing between the layers. XRD spectra of cross-linked TTCS and SiOC particles were both featureless, confirming the amorphous nature of these ceramics (hallmark of these materials). Raman spectrum (*I*_*d*_/*I*_*g*_) pre and post thermal reduction showed a slight change in accordance with previous reports (Supplementary Fig. 9)^39^. Reduction of GO to rGO was verified by the disappearance of oxide peaks in the high resolution XPS analysis of C 1*s* peak (Supplementary Fig. 10).

Thermogravimetric analysis (TGA) was performed to ascertain the mass loading of SiOC in the composite papers. Figure 1m shows the percentage composition of filtered composite paper prior to their thermal reduction. Significant weight loss was observed in the 50–100 °C and 100–400 °C temperature ranges, which is attributed to evaporation of trapped water molecules in the GO and oxygen functionalities, respectively^57^,^58^,^59^. The weight loss was highest for GO and lowest for 80SiOC (see Supplementary Table 2). Final weight loss in the 400–800 °C range is due to burning of carbon material. Comparatively, the initial weight loss was not observed in thermally reduced samples (mere 1.2% for rGO at 400 °C, Supplementary Fig. 11) that suggests high degree of water removal and oxygen groups by thermal annealing. Approximately 3% and 6–10% residue was noted for GO and rGO material at ∼800 °C. As a result SiOC content (or percentage weight remaining) in the thermally reduced composite was higher than unannealed specimens; SiOC content in 10SiOC, 40SiOC, 60SiOC and 80SiOC increased from ∼10–30%, ∼50–65%, ∼65–78% and ∼83–92%, respectively. In the traditional method of electrode preparation, active material (including recently reported graphene embedded PDC material) is mixed with polymeric binder and conductive agent in an ∼80:10:10 ratio, followed by slurry coating on metal current collector foil^47^. However, using the present method we have made a freestanding and lightweight electrode, containing up to ∼78% SiOC as active material and ∼22% of rGO (acting as binder and conductive agent). Paper electrodes were directly utilized as the working electrodes. Electrochemical performance is presented in the following section.

### Electrochemical performance

**Figure 2:**
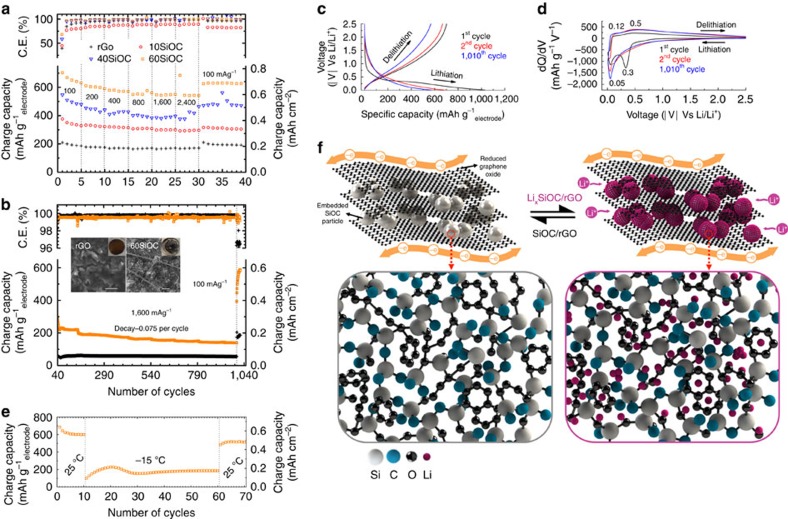
Electrochemical characteristics and proposed lithium storage mechanism. (**a**) Charge capacity and cycling efficiency of various paper electrodes when cycled asymmetrically at increasing charge current densities. (**b**) Extended cycling behavior of rGO and 60SiOC electrodes cycled symmetrically at 1,600 mA g^−1^_electrode_. After 970 cycles, the electrodes showed good recovery when the current density was lowered back to 100 mA g^−1^_electrode_. Insets show the post-cycling digital and SEM images of the dissembled rGO and 60SiOC electrodes. Scale bar is 10 μm. (**c**) Voltage profile of 60SiOC electrode and corresponding (**d**) differential capacity curves for 1st, 2nd and 1,010th cycle. (**e**) Cycling behavior of 60SiOC at sub-zero temperature. After cooling down to ∼-15 °C, the cell demonstrated a stable charge capacity of ∼200 mAh g^−1^_electrode_ at 100 mA g^−1^_electrode_. The cell regained ∼86% of its initial capacity when returned to cycling at room temperature (∼25 °C). (**f**) Schematic representing the mechanism of lithiation/delithiation in SiOC particles. Majority of lithiation occurs *via* adsorption at disordered carbon phase, which is uniformly distributed in the SiOC amorphous matrix. Large rGO sheets serve as an efficient electron conductor and elastic support.

Figure 2a shows charge capacities and columbic efficiency of rGO, 10SiOC, 40SiOC, 60SiOC electrodes asymmetrically cycled at varying charge current densities. For rGO, the first-cycle charge capacity at 100 mA g^−1^_electrode_ was ∼210 mAh g^−1^_electrode_, it dropped to ∼200 mAh g^−1^_electrode_ in the second cycle, and then the charge capacity stabilized at ∼180 mAh g^−1^_electrode_ after five cycles. When charge current density increased to 2,400 mA g^−1^_electrode_, charge capacity was retained at ∼175 mAh g^−1^_electrode_. Returning the current density back to 100 mA g^−1^_electrode_ led to the return of higher capacity of 192 mAh g^−1^_electrode_. High irreversible first-cycle capacity results from electrochemical reaction contributed to solid-electrolyte interphase (SEI) layer formation. For the composite electrode, the first-cycle charge capacity increased in correspondence to the percentage of SiOC in the electrode. For example, 10SiOC showed 376 mAh g^−1^_electrode_, while 40SiOC and 60SiOC showed 546 mAh g^−1^_electrode_ and 702 mAh g^−1^_electrode_ (volumetric capacity of ∼470 mAh  cm^−3^_electrode_), respectively. The 60SiOC capacity was lower than the capacity calculation based on a ‘rule of mixture' approach (∼793 mAh g^−1^) with constituent rGO (first cycle reversible capacity ∼210 mAh g^−1^) at ∼22 wt% as lower bound and SiOC (highest first cycle reversible capacity ∼958 mAh g^−1^ from ref. 46) at ∼78 wt% as upper bound. Similar to rGO electrode, when charge current density increased to 2,400 mA g^−1^_electrode_, composites 10SiOC, 40SiOC and 60SiOC showed high reversible capacity at 296, 417 and 543 mAh g^−1^_electrode_, respectively. Capacity retention at 2,400 mA g^−1^_electrode_ of 83.5% (compared with cycle number 5 at 100 mA g^−1^_electrode_) and first-cycle efficiency of 68% for 60SiOC is among the highest reported performances for a freestanding graphene-based electrode (see Supplementary Table 3 and Supplementary Table 4 for summary and comparison, respectively)^14^,^15^,^16^,^17^,^18^,^19^,^23^,^25^,^32^,^38^. When charge current density was lowered again to 100 mA g^−1^_electrode_ at cycle number 31, charge capacity increased to stable values of 304 mAh g^−1^_electrode_ (∼80% retained), 471 mAh g^−1^_electrode_ (∼96% retained) and 626 mAhg^−1^_electrode_ (∼97% retained) for 10SiOC, 40SiOC and 60SiOC, respectively.

In order to test cyclic stability of the electrodes, the same cells were subjected to symmetric cycling at a current density of 1,600 mA g^−1^_electrode_. Charge capacity for this test is shown in Fig. 2b. Charge capacity of 60SiOC showed some decline as the cells were subjected to prolong symmetric cycling at 1,600 mA g^−1^_electrode_. The capacity decay over the 970-cycle range was observed to be approximately 0.075 mAh g^−1^_electrode_ per cycle. This decline was not observed in the rGO specimen, thereby demonstrating the importance of graphene in the composite material. Nonetheless, the average composite paper capacity in this range was approximately three times higher than pristine rGO electrode (∼170 versus ∼58 mAh g^−1^_electrode_). Most significantly, the cell capacities were ∼185 (rGO) and 568 mAh g^−1^_electrode_ (60SiOC) at 1,010th cycle when the current density was brought back to 100 mA g^−1^_electrode_ and stabilized to 186 and 588 mAh g^−1^_electrode_, respectively at 1,020th cycle before the tests were stopped for post-cycling analysis. This represents ∼94% capacity retention for 60SiOC when compared with capacity value at the 40th cycle prior to beginning of the long-term cycling test (see Supplementary Table 3). No measureable change in cycling efficiency of 60SiOC (∼99.6%) was observed during this period. This shows that, even after 1,020 cycles, the composite electrode was robust and continued to function without appreciable degradation.

Supplementary Fig. 12a shows voltage profiles of rGO for the 1st, 2nd and 1,010th cycle. Differential capacity profiles in Supplementary Fig. 12b were similar to previous reports on rGO electrodes, with a primary reduction peak at ∼50 mV, a secondary reduction peak at ∼(520–560)  mV, and an oxidation peak at ∼(120–130)  mV^39^. The peak at ∼50 mV, present in all subsequent cycles, is associated with lithiation of graphitic carbon, whereas the peak at ∼560 mV signifies formation of SEI, which exists only in the first cycle. Supplementary Fig. 12c and d show the voltage profile and differential capacity curves of 1st and 2nd cycle of 10SiOC, respectively. The first cycle contained three reduction peaks at around ∼50, ∼240 and ∼520 mV, attributed to rGO lithiation, irreversible Li_*x*_SiOC formation, and SEI formation, respectively^39^,^41^,^45^. In contrast, only one subtle extraction peak at ∼110 mV is observed, which represents rGO de-lithiation with an extended bulge at ∼500 mV that represents Li_*x*_SiOC de-lithiation^38^,^45^,^46^,^47^. As the SiOC content increased to 40% (Supplementary Fig. 12e,f) and 60% (see Fig. 2c,d), domination of SiOC lithiation increased, as proven by increased intensity of the irreversible Li_*x*_SiOC formation peak at ∼(270–300)  mV. Peak intensity of rGO de-lithiation at ∼120 mV diminished with respect to Li_*x*_SiOC de-lithiation bulge at ∼500 mV. In addition, the 2nd and the 1,010th cycle charge/discharge and differential capacity curves of the electrodes had similar profiles, showing that no new phases formed even after more than 1,000 cycles. More importantly, the efficiency of 60SiOC remained high throughout the cycling test.

Additional rate capability test involving extreme symmetric cycling were performed on freshly prepared 60SiOC paper electrode with even higher mass loading (approximately 3 mg cm^−2^). The data is presented in Supplementary Fig. 13. Stable capacity of ∼700 mAh g^−1^_electrode_ was observed at 100 mA g^−1^_electrode_ which decreased to ∼100 mAh g^−1^_electrode_ at 2,400 mA g^−1^_electrode_ and showed complete recovery when the current density was brought back to 100 mA g^−1^_electrode_. Such stable performance is rarely reported for precursor-derived ceramic materials even on traditionally prepared electrode on copper foil where the current density and capacity are reported with respect to the active material only^46^,^47^,^48^. Tests were also conducted on 80SiOC specimen to ascertain if the charge capacity of the freestanding paper-based electrodes can be improved even further due to higher SiOC content. These attempts, however, were not successful because electrodes prepared at 80% SiOC loading were brittle and showed erratic behavior after only a few initial cycles. First-cycle charge capacity for 80SiOC was ∼762 mAh g^−1^_electrode_ and showed domination of Li_*x*_SiOC lithiation (∼330 mV) and delithiation (∼500 mV) over rGO peaks, similar to other composite electrodes (Supplementary Fig. 14a,b). The 80SiOC electrode began to demonstrate random spikes in charge capacity and efficiency with increased cycle number at high C-rate possibly due to mechanical disintegration and loss of electrical contact due to insufficient rGO loading (Supplementary Fig. 15a). Crack could be observed in the post-cycling SEM images (see Supplementary Fig. 15b–e).

Four-point electrical conductivity measurements were performed and compared for all specimens (for details, see Supplementary Note 1 and Supplementary Fig. 16). Data is summarized in Supplementary Table 5. Although average four-point resistance for 60SiOC (580 Ω) was higher than rGO paper (40 Ω), it still represents an important achievement because TTCS derived SiOC (under present pyrolysis conditions and for the given composition) was observed to be poor electrical conductor and the improved conductivity of the composite paper (5 × 10^−2^ S cm^−1^ versus ∼10^−12^ S cm^−1^ for SiOC powder^41^) is key to better C−rate characteristics. This is more evident when we compare the C−rate data for SiOC particle electrode prepared on traditional copper current collectors^46^, where the electrochemical capacity was observed to be near zero for cycling current density of 1,600 mA g^−1^.

In addition to room temperature testing, the best performing specimen (that is, 60SiOC) was subjected to electrochemical cycling at sub-zero temperature at ∼−15 °C (for details, see Supplementary Note 2). When initially cycled at room temperature (Fig. 2e), the cell had a stable charge capacity of ∼600 mAh g^−1^_electrode_ that then reduced to a stable charge capacity of ∼200 mAh g^−1^_electrode_ when cycled at low temperature. The cell regained ∼86% of its initial capacity when it returned to cycling at room temperature.

In order to verify electrode integrity, the cells were dissembled in their lithiated state and the electrode was recovered for additional characterization. The inset in Fig. 2b and Supplementary Fig. 17 show the digital photograph and SEM image of the cycled electrodes. Post-cycling Raman spectroscopy data is presented in Supplementary Fig. 18 and Supplementary Table 6. No evidence of surface cracks, volume change, or physical imperfections were observed in the SEM images, suggesting high mechanical/structural strength of the composite paper towards continuous Li-cycling which could be attributed to unique structure of the electrode as shown in Fig. 2f. In all cases, evidence of SEI formation due to repeated cycling of Li-ions was observed. Contamination in the specimen, indicated by arrows, was a result of residue of glass separator fibers. The electrodes were briefly exposed to air during the transfer process, resulting in oxidation of Li, which appeared as bright spots in the images due to non-conducting nature.

To illustrate the kinetics of charge/discharge of the composite paper, Galvanostatic intermittent titration cycling was performed for the 60SiOC electrode at room and low temperature (for details, see Supplementary Note 3). Acquired D_Li+_ varied between ∼10^−14^ and ∼10^−15^ m^2^ s^−1^ during insertion and extraction (Supplementary Fig. 19). These values are comparable with values reported for polymer-derived SiOC (Kasper *et al.* 10^−13^ to 10^−15^ m^2^ s^−1^)^44^. In addition, total polarization potential and time dependent change in open-circuit voltage (OCV) at various states of charge were inferred for these experiments, as shown in Supplementary Fig. 20a–d. Reaction resistance to Li insertion and extraction from the 60SiOC electrode was calculated by taking a ratio of OCV to the current density (Supplementary Fig. 20e,f). Reaction resistance was fairly constant at 2 Ohm  g during room temperature insertion. However, it increased exponentially to 8 Ohm g during Li extraction in the 1.5–2.0 V range, which highlights the difficulty in extracting the very last Li atoms from amorphous SiOC structure (Fig. 2f). Density of state calculations (Supplementary Fig. 21) show that Li is stored at several energy levels in the amorphous SiOC structure, with majority of the insertion occurring in the 0–0.5 V range. Further, a voltage hysteresis of ∼0.5 V exists during the extraction half, which could be attributed to the hydrogen (H-terminated edges of free carbon phase) that are generally present in the SiOC derived from thermal decomposition of organosilicon polymers. H content in pyrolyzed ceramic particles was measured to be ∼0.25–0.3 wt% (for details, see Methods section, Supplementary Fig. 2, Supplementary Table 1). Galvanostatic intermittent titration performed at low temperature (∼−15 °C) showed D_Li+_ values in the ∼(10^−15^ to 10^−13^)  m^2^ s^−1^ range during Li-ion insertion and extraction (Supplementary Fig. 22). The total polarization potential, time dependent change in OCV at various states of charge performed at ∼−15 °C and corresponding reaction resistance plots are included in Supplementary Fig. 23.

### Mechanical strength of the electrode

**Figure 3:**
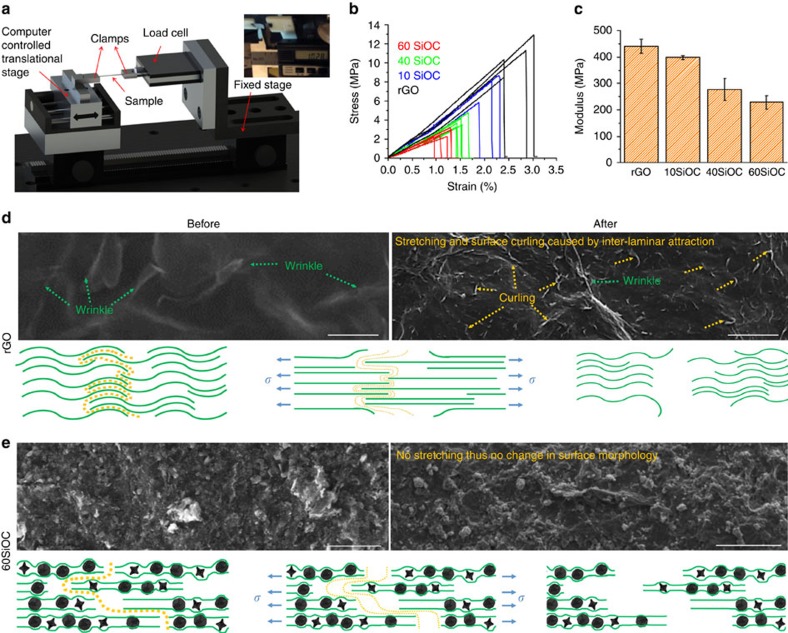
Mechanical testing data. (**a**) Schematic of the tensile testing setup with a photograph of rGO paper immediately after the fracture. Scale reading in the photograph indicate the change in length to be ∼0.28 mm. (**b**) Engineering stress versus strain plots of various freestanding papers derived from load versus displacement data, and (**c**) their corresponding modulus values. Error bars are 26.8, 7.6, 41.5, 24.1 MPa for rGO, 10SiOC, 40SiOC, and 60SiOC, respectively. The SEM images and schematic illustration to show the predicted mechanism of fracture in rGO and 60SiOC freestanding papers: (**d**) The rGO paper experienced stretching and rearrangement of graphene sheets before failure. (**e**) For 60SiOC paper, negligible stretching or rearrangement occurred. Fracture line follows SiOC particles embedded in rGO flakes, resulting in gradual separation/tearing of the paper. The scale bar is 20 μm in all images. Tensile test videos are included as Supplementary Movies 1 and 2.

Static uniaxial tensile tests were conducted to quantify the strength and strain-to-failure for the freestanding composite papers by use of a custom-built set-up. Figure 3a shows a schematic of the test setup, in which the load cell is attached to a digital meter, connected to a transducer electronic data sheet in order to transfer the data to host computer through an RS232 serial port using a program written in MATLAB. Engineering stress–strain plots and tensile modulus, derived from load–displacement curves for various paper electrodes are compared in Fig. 3b,c, respectively. The rGO sample showed average tensile strength of ∼10.7 MPa at a failure strain of 2.8%, while 60SiOC sample had tensile strength of ∼2.7 MPa at a strain of 1.1%. Low tensile strength of the 60SiOC specimen was expected considering that it contained only ∼20% rGO. Overall, strength and modulus for these crumpled composite papers was lower than GO and rGO papers prepared from techniques other than high temperature reduction^53^,^54^. However, the strain-to-failure was almost 5 to 10 times higher than a typical GO, rGO or rGO-composite paper, suggesting that crumpled composite papers may be able to sustain larger volume changes. Surface analysis using SEM of rGO (Fig. 3d) showed occurrence of micro features after tensile test, which we suggest, are due to rearrangement of rGO sheets under tensile load. These micro features are assumed to be due to curling of individual graphene sheets on the top surface when they lose contact with the sheets below them. However, for 60SiOC in Fig. 3e, ceramic particles acted as the point of fracture and caused rGO sheets to separate without stretching, as proven by SEM images that show no distinguishable changes before and after tensile test. Supplementary Fig. 24a–h are the top and cross-sectional view SEM images of fractured surface. The rGO because of higher elasticity had an irregular crumpled appearance, but composite papers were more brittle and had sharper cross-section. Mode of fracture in rGO and 60SiOC papers differed significantly, as presented in Supplementary Movies 1 and 2. A loud distinct sound indicated almost instantaneous fracture of the rGO specimen, accompanied by curling of both ends of the fractured paper. Fracture of 60SiOC specimen was similar to a thin plate with an edge crack, the crack propagation could be clearly observed. In addition, stress lines could be observed only in the rGO specimen, radiating from one clamp to another and indicating distribution of stress throughout the length of the specimen. These observations are explained with the help of a schematic in Fig. 3d,e. *Ex situ* Raman analysis (Supplementary Fig. 25) from the top surface of the specimens before and after tests showed increase in average intensity ratio of the *I*_*d*_ and *I*_*g*_ peaks for rGO (0.88 versus 1.02) while the ratio was largely unaffected for composite specimen.

## Discussion

Electrochemical characterization shows that 60SiOC is best long-term cycling electrode with reversible capacities of ∼702 mAh g^−1^_electrode_ at 1st cycle and ∼588 mAh g^−1^_electrode_ at 1,020th cycle, respectively. Although 80SiOC offers highest first reversible capacity of ∼762 mAh g^−1^_electrode_, it undergoes capacity fading and mechanical damage after few initial cycles at high currents. Hence, the capacity and cycling stability are affected by the relative amounts of SiOC and graphene in the composite, respectively. We ascribe the superior electrochemical performance of 60SiOC electrode to remarkable physical and chemical properties of its constituents and the unique morphological features of the paper. Because graphene sheets in 60SiOC occupy larger volume in the composite, well-dispersed GO sheets during the layer−by−layer filtration process arrange themselves around the SiOC particles to form a flexible composite paper. TEM (Supplementary Fig. 6), SEM (Supplementary Fig. 7) and FIB (Supplementary Fig. 8) characterization shows that morphology of the composite paper is planar and porous. The porous design therefore facilitated liquid electrolyte to reach the very interior of the electrode thereby providing easy path for solvated ions to be transported on to the surface of SiOC particles. Further, rGO because of its high electrical conductivity and mechanical flexibility provided an electrically conducting (see Supplementary Table 5) and mechanically robust (see Fig. 3b) matrix for the Li-active SiOC particles thereby buffering volume changes in the electrode and maintaining inter particle connection during long-term cycling. Microscopy (Fig. 2b, Supplementary Fig. 17) and Raman spectroscopy (Supplementary Fig. 18) of the disassembled cell reveal formation of stable SEI on a completely integral electrode, which could explain the high cycling efficiency observed in these composites.

We attribute high reversible capacity of molecular precursor derived SiOC to its amorphous structure, which is comprised of silica domains, –*sp*^2^ carbon chains (or the free carbon phase), nano-voids and silicon/carbon open bonds (Fig. 2f and Supplementary Fig. 4 for proposed SiOC structure), that offer large number of sites, in which Li-ion can be reversibly stored. We notice that even the composite electrodes are not free from charge–discharge voltage hysteresis (or energy inefficiency) that is generally observed in precursor derived ceramics during the extraction half^46^,^49^,^50^. Lowering hydrogen content^60^ and doping of silica domains (such as B) in SiOC could be a useful strategy for improving electrical properties and lowering of voltage hysteresis in these ceramics^40^,^46^. Another important area for future investigation could be to tailor the rGO flakes for residual oxygen and hydrogen surface groups and edge defects so that lithium irreversibility and voltage hysteresis^60^ that arises from active defect sites could be minimized without compromising Li-ions' mobility and access to the SiOC particles^4^.

In summary we have demonstrated fabrication of a freestanding multi-component composite paper consisting of SiOC glass-ceramic particles supported in rGO matrix as a stable and durable battery electrode. The porous 3-D rGO matrix served as an effective current collector and electron conductor with a stable chemical and mechanical structure while, embedded amorphous SiOC particles actively cycled Li-ions with high efficiency. Elimination of inactive ingredients such as metal current collector, non-conducting polymeric binder and conducting agent reduces the total electrode weight and provides the means to produce highly efficient lightweight batteries.

## Methods

### Preparation of polymer derived SiOC ceramic

SiOC was prepared through the polymer pyrolysis route^41^, liquid 1,3,5,7-tetramethyl-1,3,5,7-tetravinylcyclotetrasiloxane (TTCS, Gelest, PA) precursor (with 1 wt% dicumyl peroxide added as the cross-linking agent) was cross-linked at 380 °C in argon for 5 h, which resulted in a white infusible mass. The infusible polymer was ball-milled in to fine powder and pyrolyzed at 1,000 °C for 10 h in flowing argon resulting in a fine black SiOC ceramic powder.

### Chemicals

Sodium nitrate (99.2%), potassium permanganate (99.4%), sulfuric acid (96.4%), hydrogen peroxide (31.3% solution in water), hydrochloric acid (30% solution in water) and methanol (99.9%) were purchased from Fisher Scientific. All materials were used as received without further purification.

### Preparation of GO and SiOC composite paper

Modified Hummer's method was used to make GO^51^. A total of, 20 ml colloidal suspension of GO in 1:1 (v/v) water and isopropanol was made by sonication. Varying weight percentages of SiOC particles (with respect to GO) were added to the solution and the solution was sonicated for 1 h and stirred for ∼6 h for homogenous mixing. The composite suspension was then filtered by vacuum filtration through a 10 μm filter membrane (HPLC grade, Millipore). The GO/SiOC composite paper obtained was carefully removed from the filter paper, dried, and thermally reduced at 500 °C under argon atmosphere for 2 h. The large-area paper with 60SiOC composition (with an ∼6.25 inch diameter, cut into rectangular strip) was similarly prepared by use of a Büchner funnel with a polypropylene filter paper (Celgard). The heat-treated paper was then punched (cut) into small circles and used as working electrode material for Li-ion battery half-cells.

### Coin cell assembly and electrochemical measurements

Li-ion battery coin cells were assembled in an argon-filled glove box. 1 M LiPF_6_ (Alfa Aesar) in (1:1 v/v) dimethyl carbonate:ethylene carbonate (ionic conductivity 10.7 mS cm^−1^) was used as the electrolyte. A 25 μm thick (19 mm diameter) glass separator soaked in electrolyte was placed between the working electrode and pure Li foil (14.3 mm diameter, 75 μm thick) as the counter electrode. Washer, spring, and a top casing were placed to complete the assembly before crimping.

Electrochemical performance of the assembled coin cells was tested using a multichannel BT2000 Arbin test unit sweeping between 2.5 V to 10 mV versus Li/Li^+^ that followed a cycle schedule: (a) Asymmteric mode: Li was inserted at 100 mA g^−1^_electrode_, while the extraction was performed at increasing current densities of 100, 200, 400, 800, 1,600 and 2,400 mA g^−1^_electrode_ for 5 cycles each, and returned to 100 mA g^−1^_electrode_ for the next 10 cycles. (b) Symmetric mode: later, all the cells were subjected to symmetric cycling at a current density of 1,600 mA g^−1^_electrode_ for up to 1,000 cycles, returning to 100 mA g^−1^_electrode_ for the last 20 cycles.

### Instrumentation and characterization

SEM of SiOC powder was carried out on a Carl Zeiss EVO MA10 system with incident voltage of 5–30 kV. TEM images were digitally acquired by use of a Phillips CM100 operated at 100 kV. TEM elemental mapping was performed by using a 200 kV S/TEM system (FEI Osiris) equipped with chemiSTEM technology, a high angle annular dark field (HAADF) and Super-X windowless EDX detector. Super-X windowless EDX detector system with silicon drift detector technology allowed fast EDX data collection (a factor of more than 50 enhancement in acquisition speed of EDX chemical mapping) and large field of view elemental mapping. Acceleration voltage was 200 kV and acquisition time was 10 min.

A FIB system (FEI Versa 3D Dual Beam) was used for milling and imaging cross-section of the paper electrodes following standard procedures. Briefly, a platinum protective layer (∼25 μm × 10 μm × 5 μm in x, y and z axes, respectively) was first deposited at an ion beam current of ∼5 nA. Milling was then performed using regular cross section at an ion beam current of ∼65 nA to create trenches on either side and bottom face of platinum-coated area. Followed by cleaning cross-section feature (∼20 μm × 1 μm × 6 μm in x, y and z axes, respectively) to fine mill contamination at the bottom face of platinum coated area. The acceleration voltage of Ga^+^ was 30 kV. An ion-beam current of ∼40 pA was used for imaging purposes. In-column detector for secondary electrons in beam deceleration mode was used for SEM imaging of the milled cross-section. Elemental mapping (EDS) was performed by use of an inbuilt energy dispersive spectroscopy silicon drift detector (Oxford Instruments).

Raman spectra were collected using a confocal Raman imaging system (Horiba Jobin Yvon LabRam ARAMIS) with 633 nm HeNe laser (laser power of 17 mW) as the light source with a × 100 microscope objective. Data acquisition was performed at an exposure time of 20 s with at least four accumulations at each point. D1 filter (10% transparency) was employed for the ceramic powder samples. Additional material characterization was made using XRD operating at room temperature, with nickel-filtered CuK*α* radiation (*λ*=1.5418 Å). The surface chemical composition was studied by XPS (PHI Quantera SXM-03 Scanning XPS Microprobe) using monochromatic Al K*α* radiation. For XPS depth profiling, sputtering was performed with a 5 keV Argon ion gun for 20 min followed by survey scan. The sputtered area was set to ∼2 mm × 2 mm. The process was repeated four times with total sputtering time reaching 80 min.

Further, bulk elemental composition of the pyrolyzed SiOC ceramic was measured following procedures similar to as described in the literature^46^. Analysis was done for carbon, oxygen and hydrogen content. Silicon content was calculated as a difference to 100%. The carbon content was measured by use of LECO Analyzer Model CS844 (LECO Corp. Analytical Bus, St Joseph, MI) by the combustion method and IR detection. Approximately 50 mg of SiOC powder mixed with accelerants as Iron chips and Lecocel II HP was used for this test. The oxygen and hydrogen contents were measured by use of LECO Analyzer Model No. ONH-836 (LECO Corp. Analytical Bus, St Joseph, MI) based on inert gas fusion thermal conductivity/infrared detection method. Specimen preparation involved mixing ∼34 mg of SiOC ceramic powder with graphite powder (LECO Corp.) as an accelerant in a nickel capsule (LECO Corp.) followed by placement in graphite crucible. The crucible was then heated to ∼3,000 °C in the chamber and gaseous products transferred to IR/thermal conductivity detectors for analysis. The mass per cent of carbon and oxygen were quantified in reference to the IR spectrum generated from graphite and tungsten oxide powders, respectively.

Hydrogen content in SiOC ceramic was also confirmed by use of another equipment based on combustion/thermal conductivity detector method, CE-440 Elemental Analyser (Exeter Analytical, UK). Combustion of the weighed sample (1.8056, mg of fine powder) was carried out in the instrument chamber in pure oxygen under static conditions. Helium carried the combustion products through the analytical system to atmosphere. Between the thermal conductivity cells absorption trap removed water from the sample gas. The differential signal read before and after the trap reflected the water concentration and, therefore, the amount of hydrogen in the original sample. The hydrogen content by this method was observed to be 0.25 wt% with an error of 0.06%. TGA was performed using Shimadzu 50 TGA (limited to 800 °C). Samples weighing, ∼2.5 mg, were heated in a platinum pan at a rate of 10 °C min^−1^ in air flowing at 20 ml min^−1^. Electrical conductivity measurements were carried out by use of a four-point probe setup and Keithley 2636A (Cleveland, OH) dual channel sourcemeter in the Ohmic region. Electrochemical cycling of assembled cells was carried out using multichannel Battery Test Equipment (Arbin-BT2000, Austin, TX) at atmospheric conditions.

### Mechanical testing

Static uniaxial in-plane tensile tests were conducted in a custom-built test setup. One end of the setup was connected to a 1N load cell (ULC-1N Interface) and the other end was clamped to a computer-controlled translation stage (M-111.2DG from PI). The entire setup was located on a bench with self-adjusting feet. All tensile tests were conducted in controlled strain rate mode with a strain rate of 0.2% min^−1^. Paper electrodes were cut (punched out) into rectangular strips of ∼5 × 15 mm^2^ for testing without any further modification.

## Author contribution

L.D. prepared all composite specimens, performed electrochemical testing, raman spectroscopy, low magnification TEM and mechanical testing. U.B. assisted L.D. with cell assembly. R.B. synthesized SiOC particles. G.S. conceived the idea, designed the experiments, performed elemental analysis/mapping and wrote the manuscript with inputs from L.D. All authors discussed the results and commented or revised the manuscript.

## Additional information

**How to cite this article:** David, L. *et al.* Silicon oxycarbide glass-graphene composite paper electrode for long-cycle lithium-ion batteries. *Nat. Commun.* 7:10998 doi: 10.1038/ncomms10998 (2016).

## Supplementary Material

Supplementary Information Supplementary Figures 1-25, Supplementary Tables 1-6, Supplementary Notes 1-3 and Supplementary References

Supplementary Movie 1 Tensile test video of rGO paper electrode

Supplementary Movie 2 Tensile test video of 60SiOC composite paper electrode
