# Supplementary material for: Silicon oxycarbide glass-graphene composite paper electrode for long-cycle lithium-ion batteries
Source: Nat Commun. 2016 Mar 30;7:10998. doi: 10.1038/ncomms10998 (PMC4820847; doi:10.1038/ncomms10998)
Supplement: Supplementary Information — Supplementary Figures 1-25, Supplementary Tables 1-6, Supplementary Notes 1-3 and Supplementary References [file ncomms10998-s1.pdf]

## Supplementary Figures

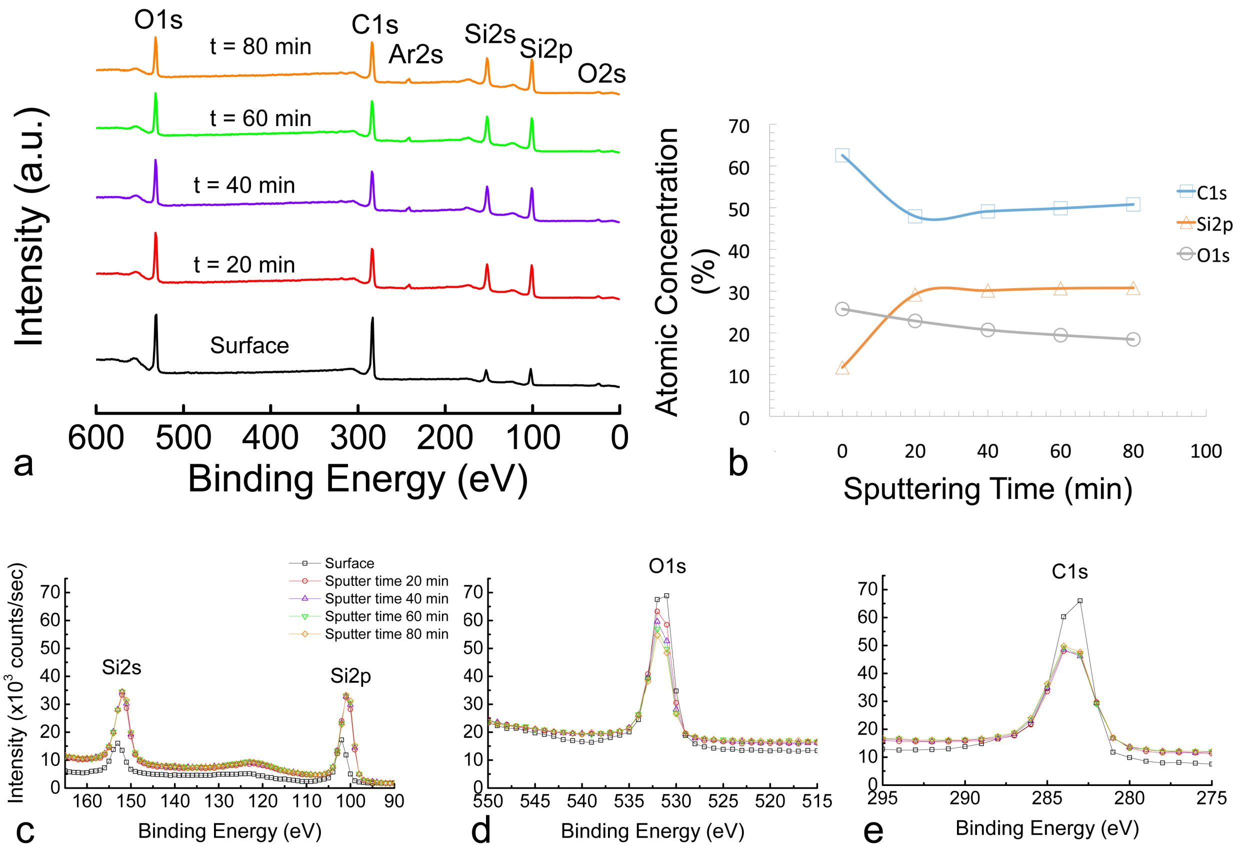


**SUPPLEMENTARY FIGURE 1: XPS analysis of SiOC particles.** (a) XPS survey scan of SiOC surface and after etching at 20 min intervals. Sputtering performed with Ar-ions at 5 keV. (b) Relative atomic concentration of each constituent from the survey scans versus sputter time. (c-e) Magnified view of XPS spectra of Si, O, and C after each etching time.

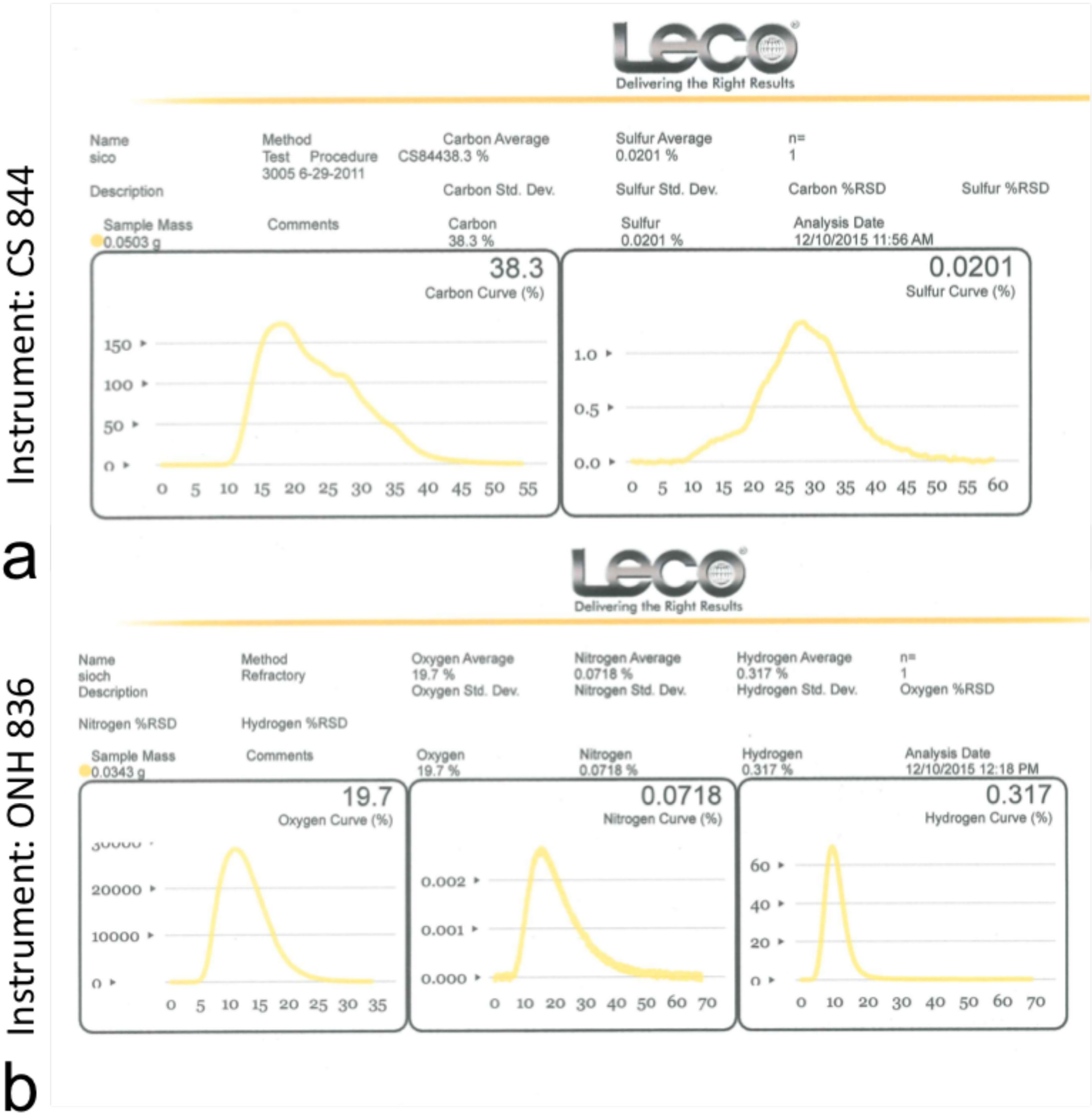


**SUPPLEMENTARY FIGURE 2: SiOC elemental composition data.** (a) Combustion (carbon) and (b) Fusion technique (oxygen, hydrogen). All values in weight percentage and axes are arbitrary units. Negligible amounts of S and N were detected (most likely due to contamination).

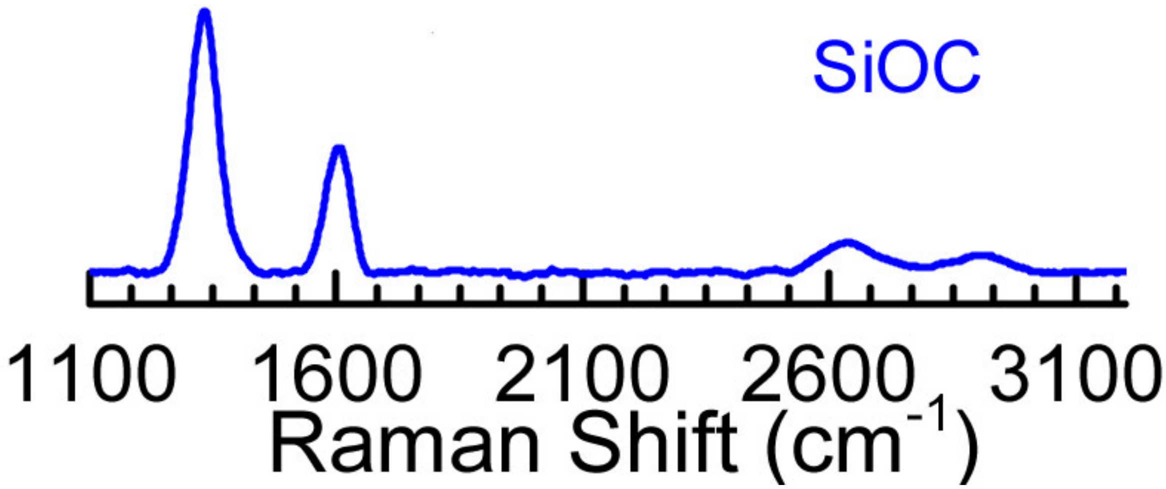


**SUPPLEMENTARY FIGURE 3: Full range Raman spectrum for SiOC particles.** Bumps at 2640 and 2915 $cm^{-1}$ are attributed to 2D and D+G modes, respectively.

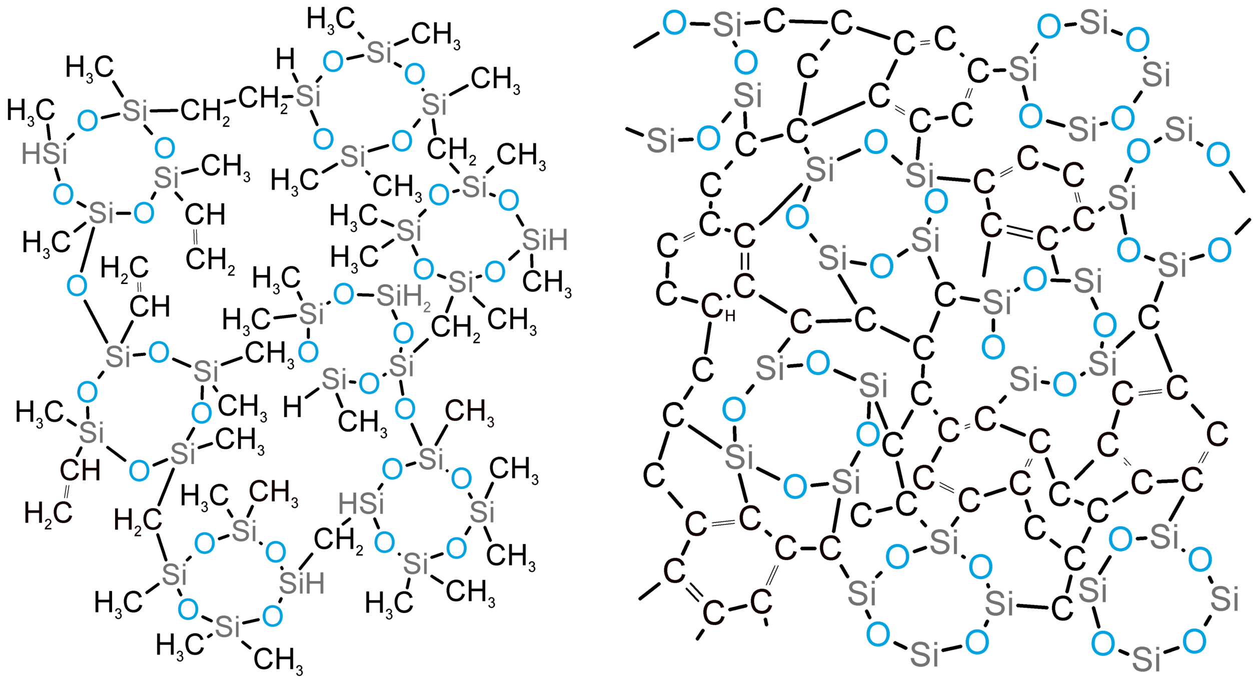

a: cross-linked b: pyrolyzed at 1000 °C

**SUPPLEMENTARY FIGURE 4:** Schematic illustration showing the predicted structure of (a) cross-linked polymeric precursor (i.e. TTCS) and (b) SiOC ceramic upon pyrolysis at 1000 °C. Polymer to ceramic transformation involves elimination of organic groups and rearrangement of carbon atoms (generally those attached to Si/O ring structure). This type of rigid network consisting of Si mixed bond tetrahedral with C and O along with free carbon phase is plausible based on the Raman, FTIR, XRD, and XPS data presented here and previous work on similar systems.

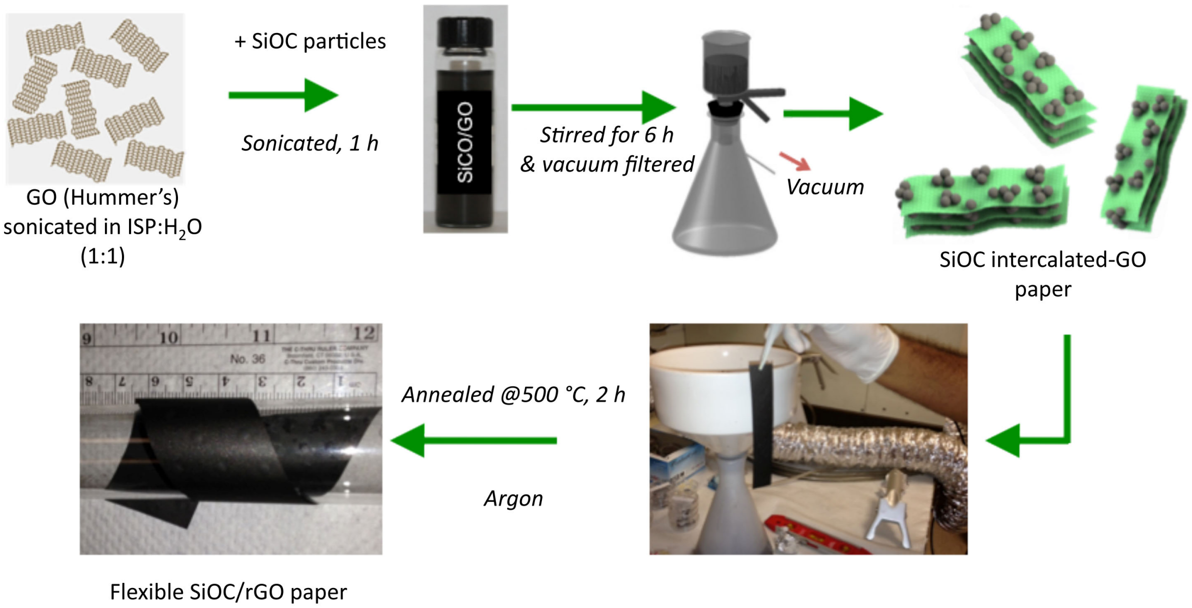


**SUPPLEMENTARY FIGURE 5: Electrode design and fabrication.** Schematic illustrating the procedure for fabrication of freestanding SiOC/graphene composite paper for use as Li-ion battery electrode. The composite paper was sufficiently robust to be handled by hand and flexible enough to be rolled on to a cylinder several times without breaking.

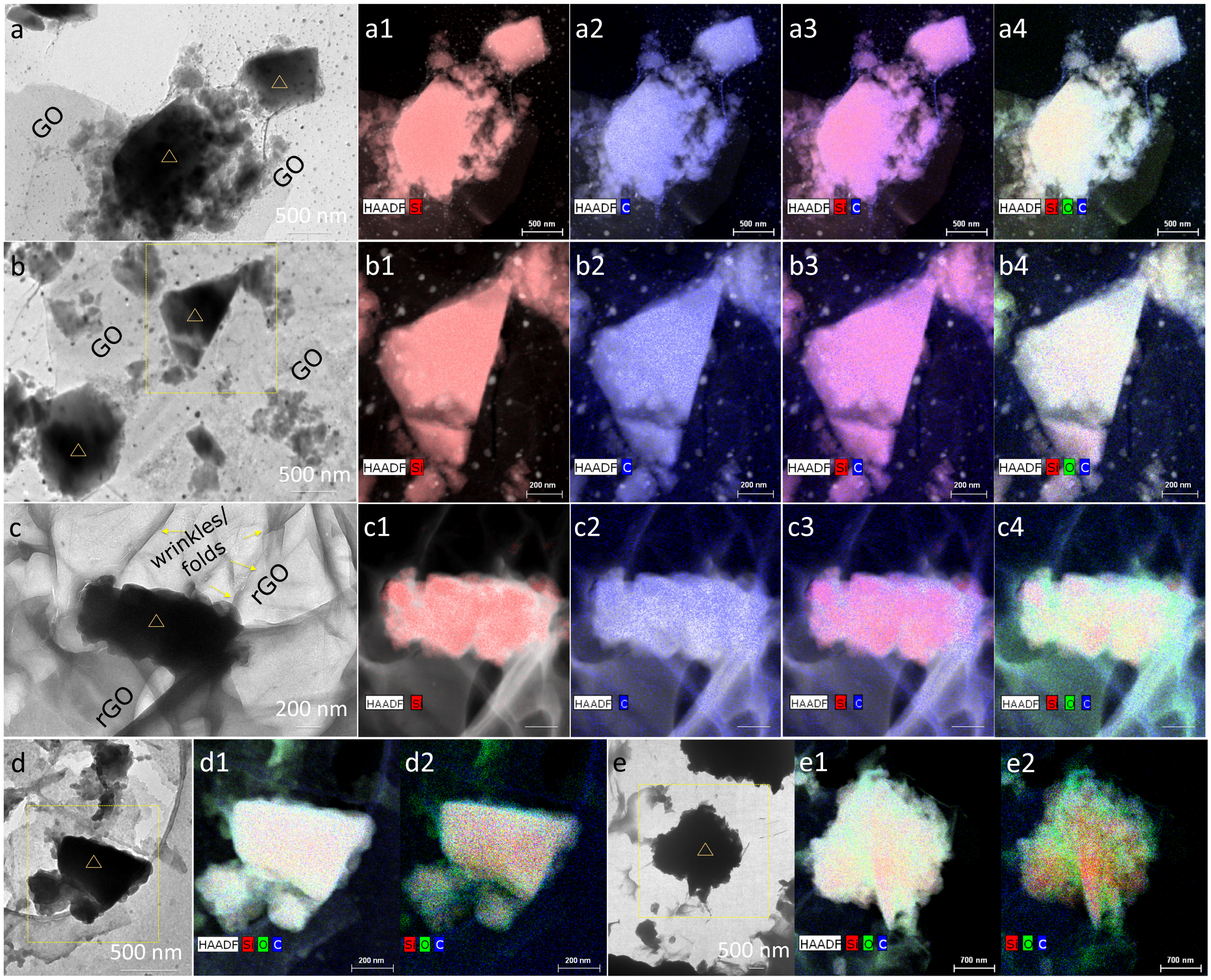


**SUPPLEMENTARY FIGURE 6: TEM elemental mapping.** (a, b) TEM images of SiOC/GO material. Micrometer size SiOC particles (Δ symbol) surrounded by nanometer size SiOC particles and interlaced or covered by large GO flakes could be observed. (a1 to a4, b2 to b4) are corresponding elemental mapping images obtained via EDX analysis in Scanning TEM mode. (c, d, e) TEM images of SiOC/rGO material (specimen prepared by sonicating annealed 60SiOC paper in propanol). (c1 to c4, d1 to d2, e1 to e2) are corresponding elemental mapping images. Uniform distribution of Si, C, and O was observed in the interior of the particle while increased accumulation of C was noted along the particle edges. HAADF: high angle annular dark field, Si: red, C: blue, O: green. Scale bar in c1 to c4 is 200 nm.

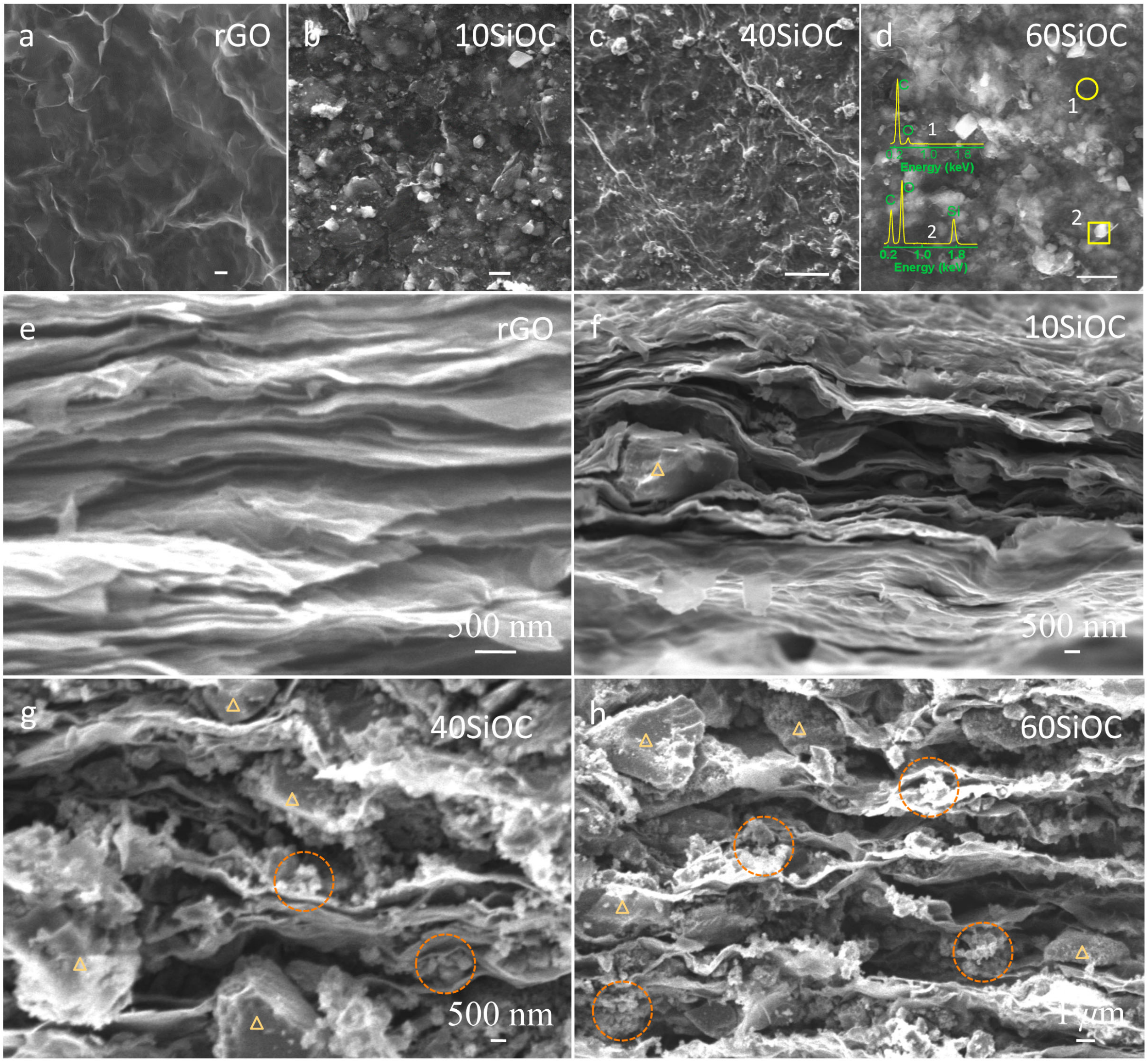


**SUPPLEMENTARY FIGURE 7: Structure of thermally reduced rGO and composite papers.** SEM top view images of (a) rGO, (b) 10SiOC, (c) 40SiOC and (d) 60SiOC paper (inset: EDX from Spot 1 showed peaks at 0.27 keV and 0.52 keV, corresponding to C: 90 at% and O: 10 at% Kα energy, respectively. In addition to C and O peaks, another peak at 1.75 keV, corresponding to Si Kα energy, was observed from Spot 2). The particle had EDX surface composition of 17 at% Si, 46 at% O and 37 at% C. Scale bar is 10 μm. (e-h) are the corresponding SEM cross-sectional images of fractured specimens.

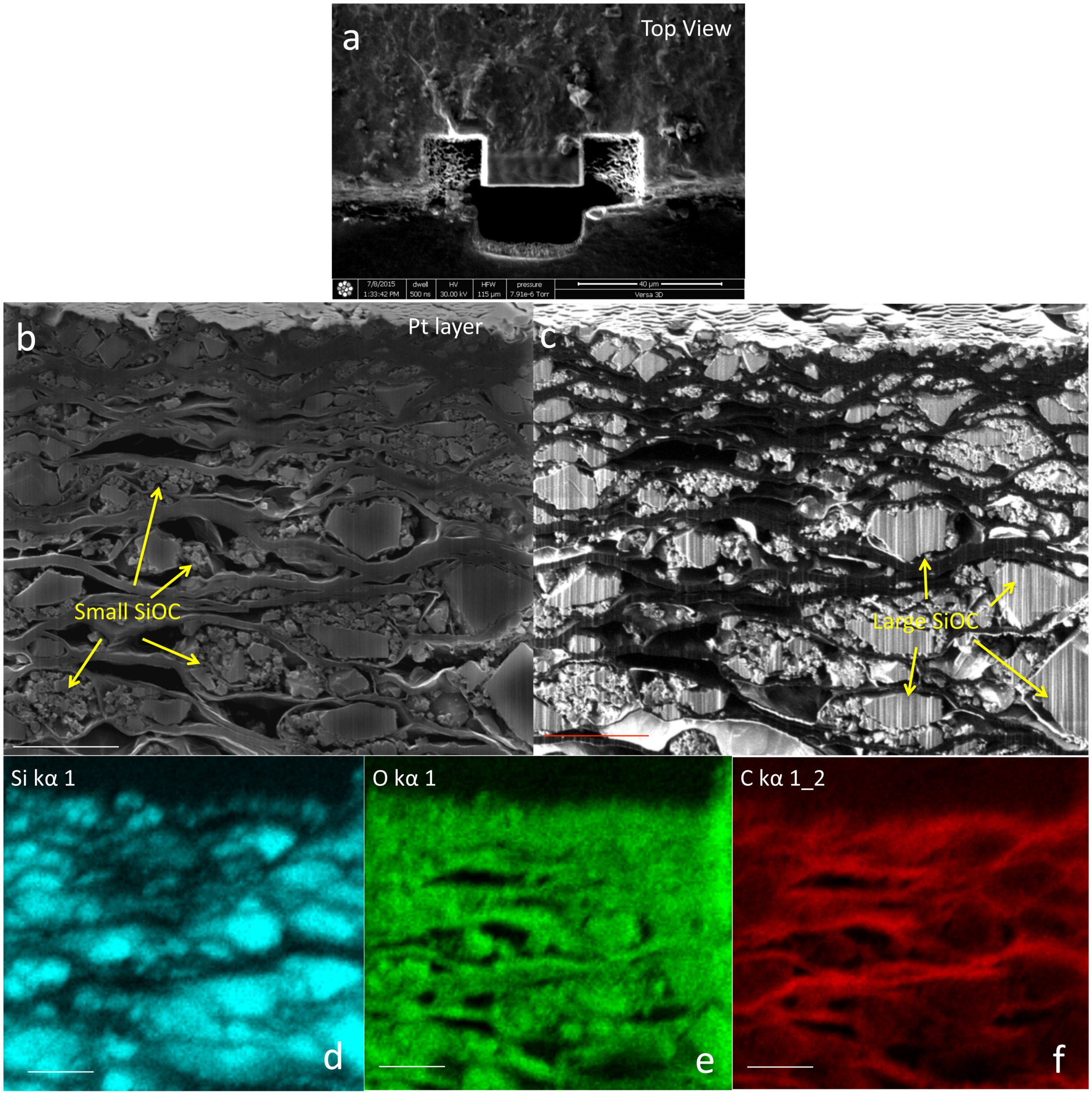


**SUPPLEMENTARY FIGURE 8: FIB cross-section images of 60SiOC composite paper (post-annealing).** (a) FIB top view showing the Pt deposit and Ion-milled trenches (scale bar is 40 μm). (b) Electron beam and (c) Ion beam images of the cross-section. Both small (nano) and large (micrometer) SiOC particles wrapped with rGO platelets could be observed. (d-f) SEM-EDX color maps showing distribution of Si, O, and C elements (scale bar is 10 μm).

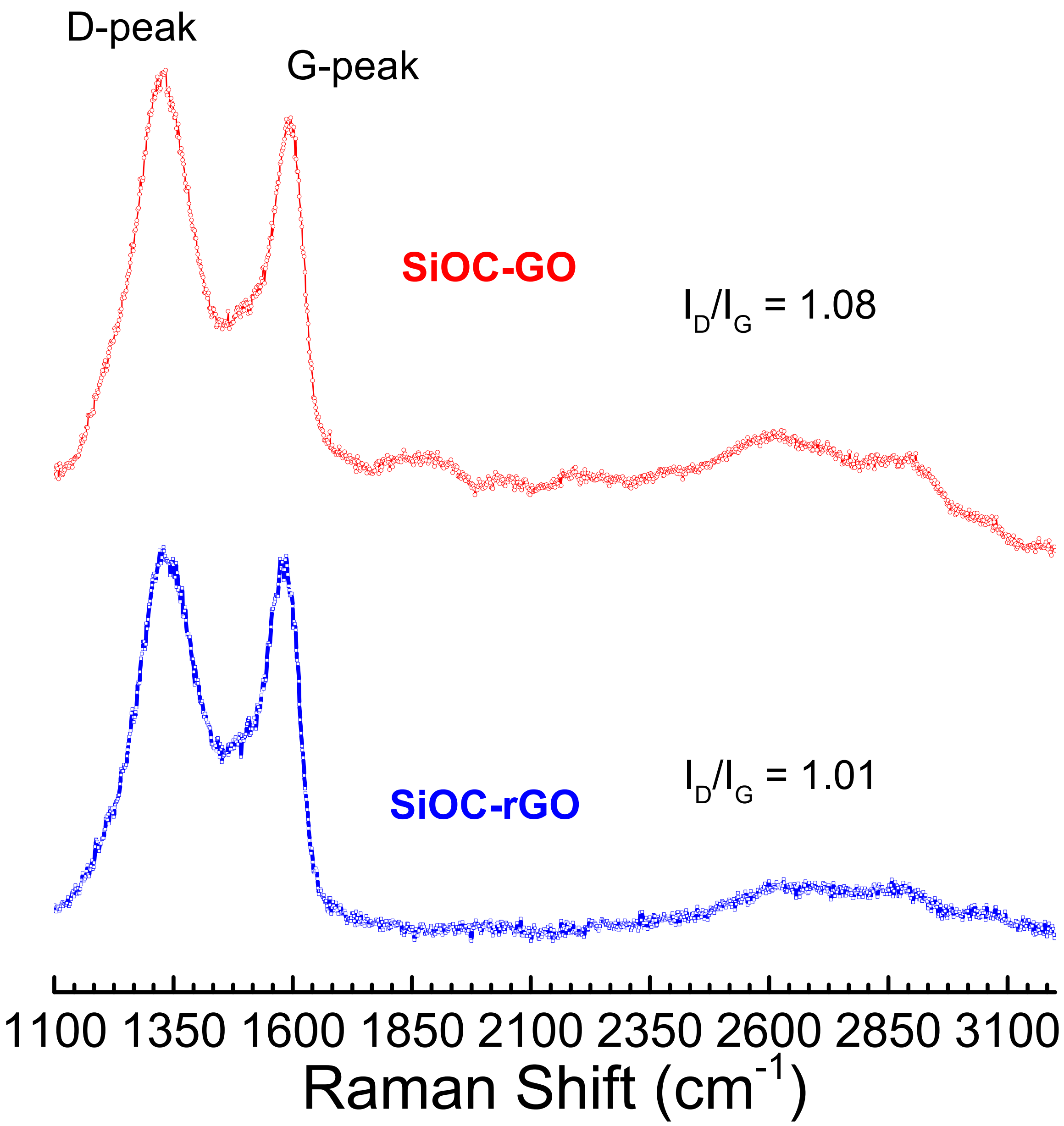


**SUPPLEMENTARY FIGURE 9: Raman spectrum of 60SiOC composite paper.** Comparison of Raman spectra of freestanding papers before and after annealing showed no distinct change in position while a slight change in relative intensity of the $I_D/I_G$ peaks was observed (~1.08 to 1.01).

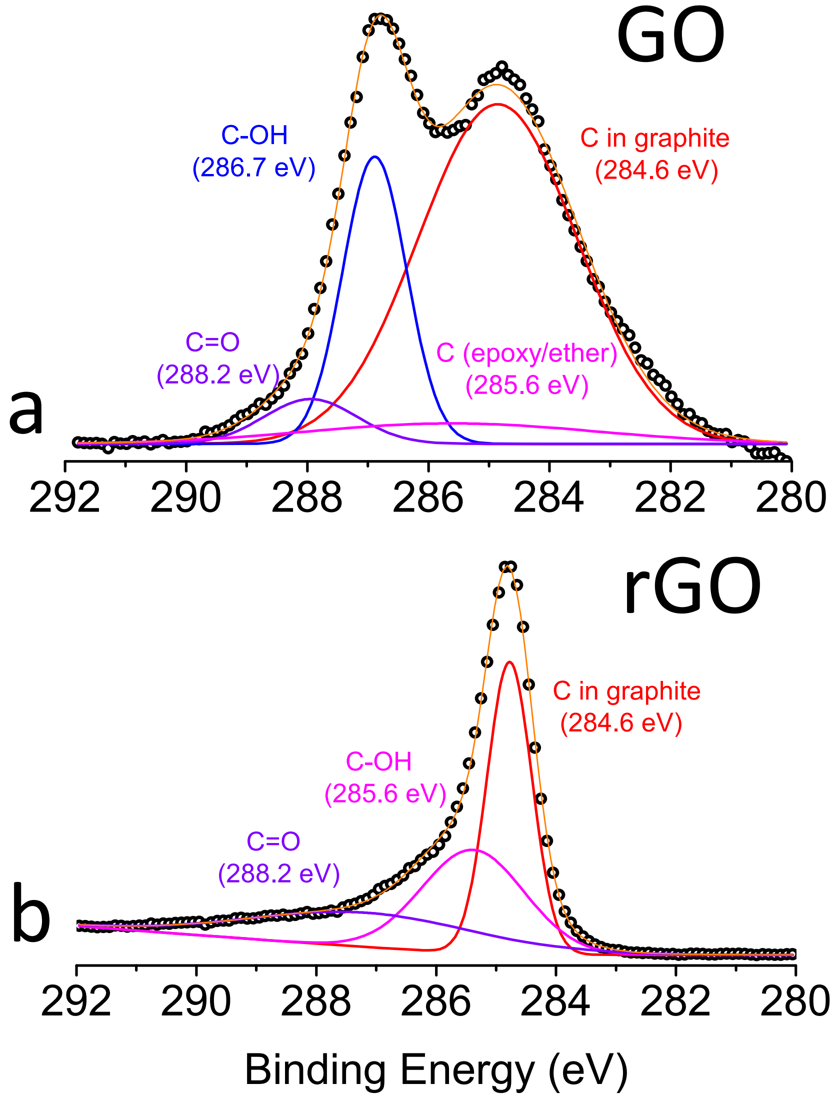


**SUPPLEMENTARY FIGURE 10: XPS before and after thermal reduction.** Deconvoluted XPS spectrum of (a) as-prepared (GO) and (b) reduced (rGO) paper in the C 1s region, the C-OH peak at 286.7 eV completely disappeared upon annealing proving the reduction of GO to rGO.

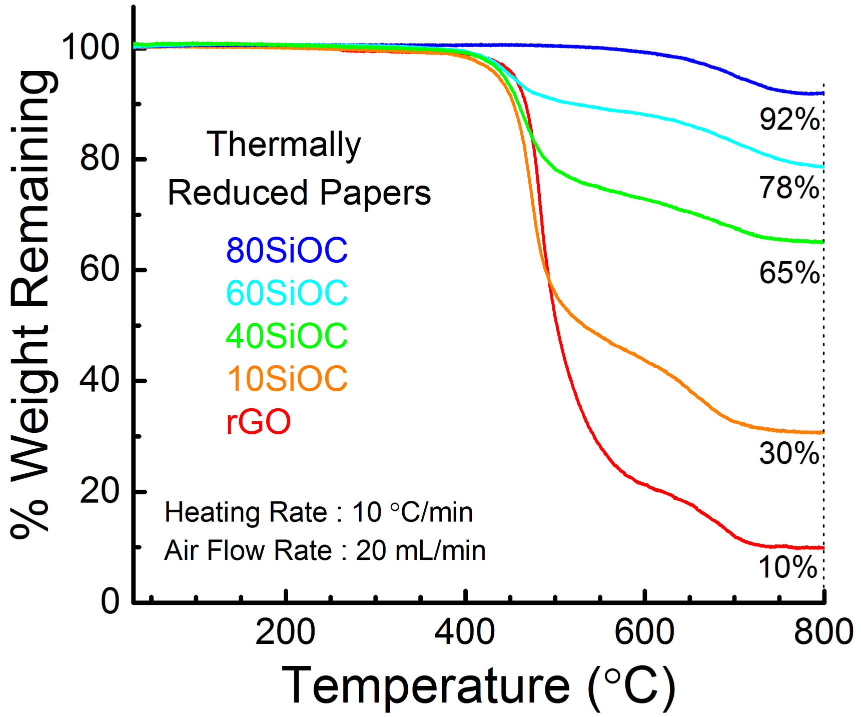


**SUPPLEMENTARY FIGURE 11: TGA of thermally reduced papers.** TGA curves of rGO paper and composite papers measured from 30 to 800 °C at a heating rate of 10 °C $min^{-1}$ in air. The weight percentage of SiOC in the composite paper is as indicated in the figure.

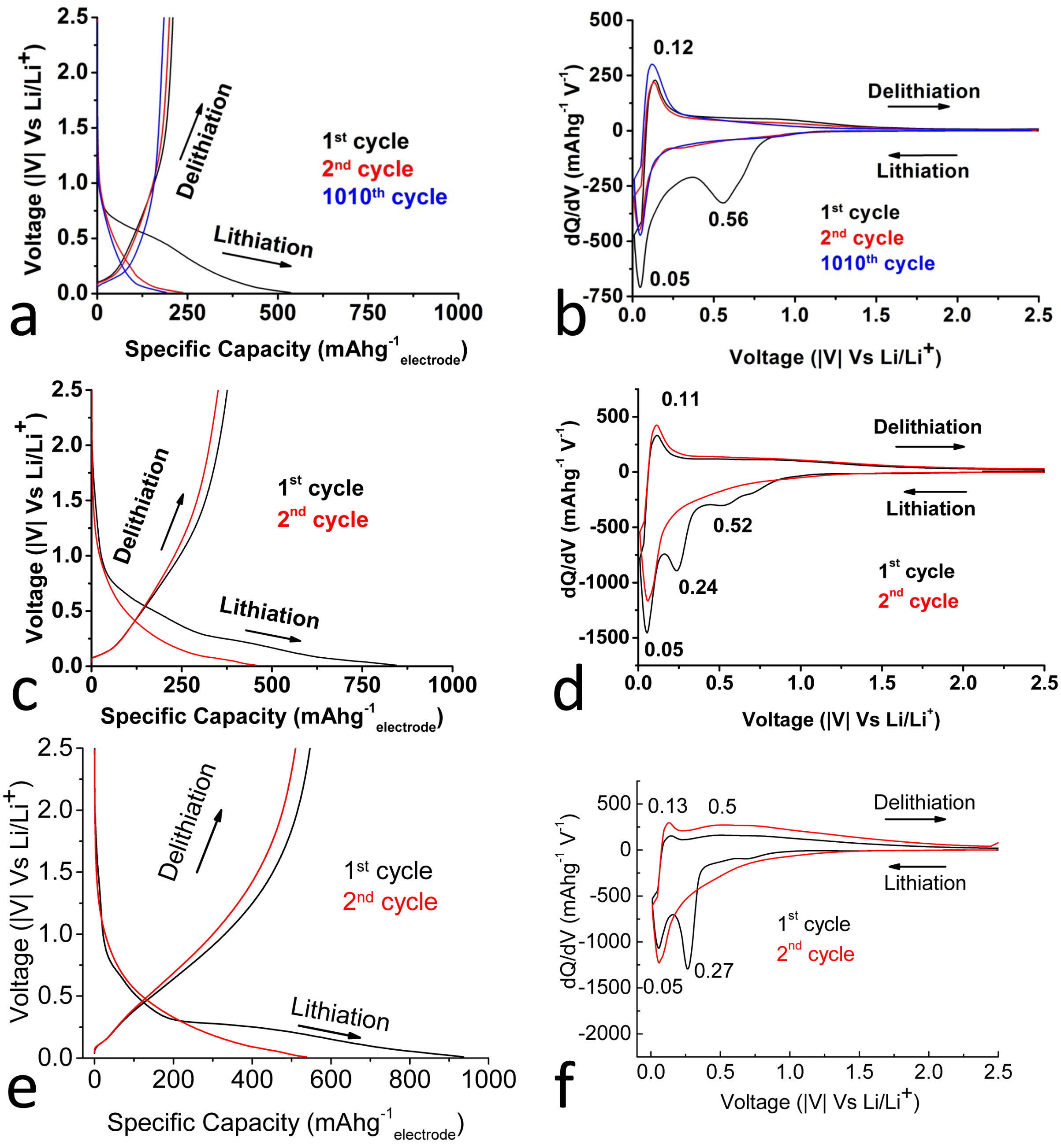


**SUPPLEMENTARY FIGURE 12: Voltage profiles along with corresponding differentiated capacity curve.** (a, b) rGO, (c, d) 10SiOC, and (e, f) 40SiOC. Lithiation peak at ~0.5 V and delithiation peak at ~0.1 V are distinct to Li reaction with rGO while peak at ~ 0.24 V is distinct to Li reaction with SiOC in the first cycle.

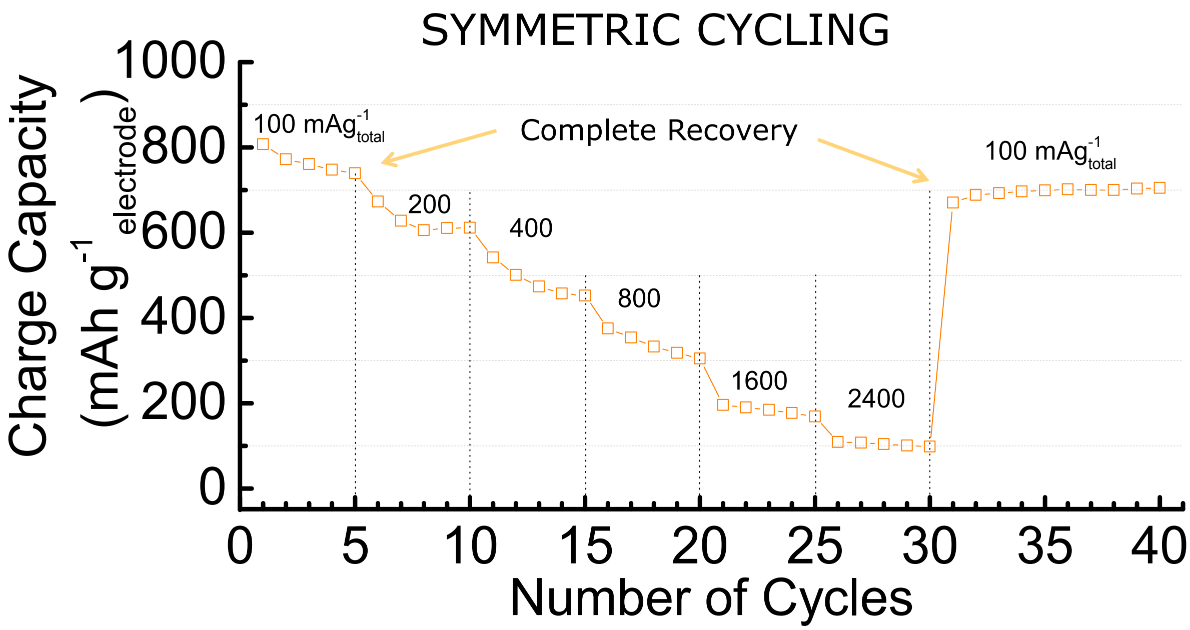


**SUPPLEMENTARY FIGURE 13:** Symmetric rate capability data for 60SiOC electrode.

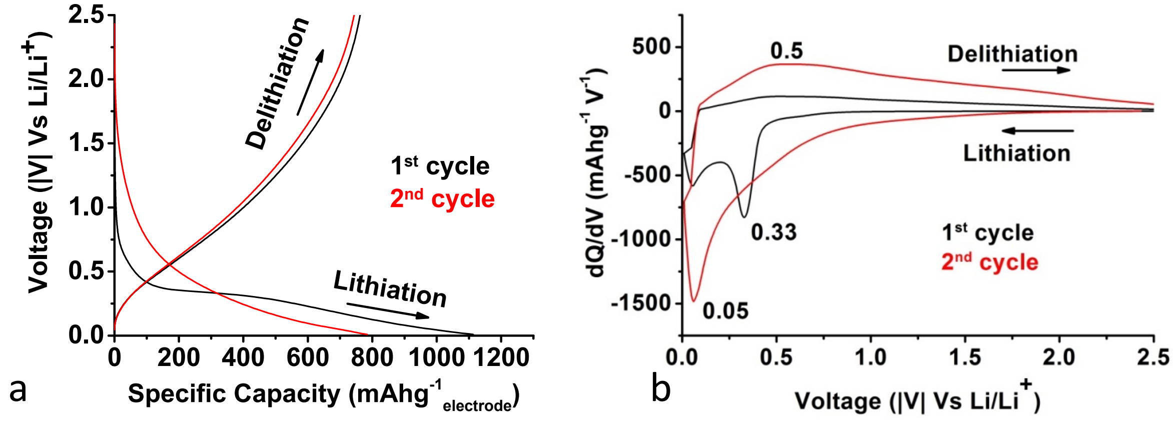


**SUPPLEMENTARY FIGURE 14: Electrochemical data for the 80SiOC electrode.** (a) Voltage profiles along with corresponding (b) differentiated capacity curve. The peaks at ~0.3 and 0.05 V in the differentiated capacity curve are distinct to Lithium's reaction with SiOC. The Li/rGO interaction peak is virtually absent in the first cycle curve.

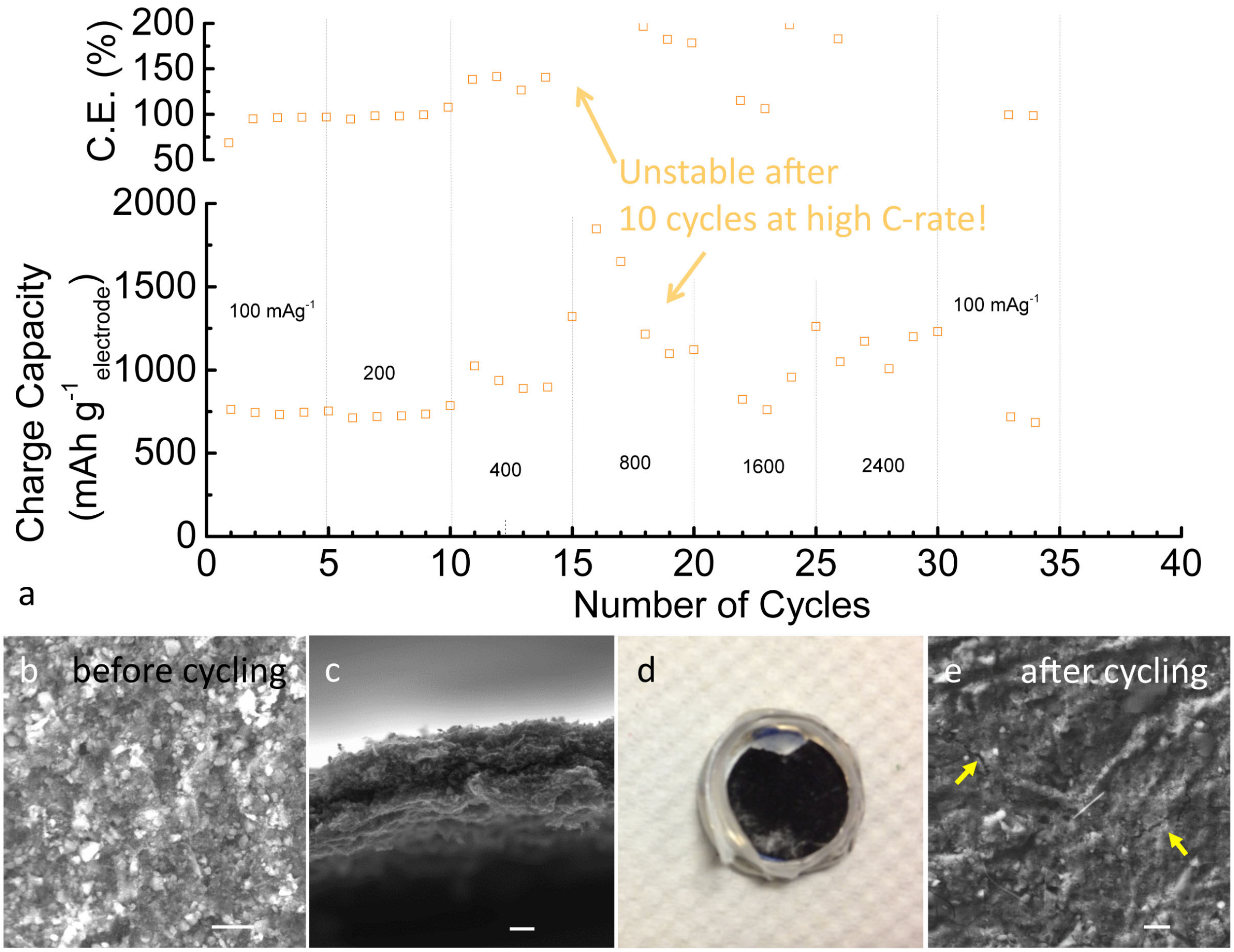


**SUPPLEMENTARY FIGURE 15: Electrochemical data for the 80SiOC electrode and post-cycling images.** (a) Charge capacity when cycled at different rates along with their cyclic efficiencies. Even though the initial capacity was high at ~762 mAh $g^{-1}_{electrode}$, the electrode began to disintegrate as the current density was progressively increased. This was expected, as there was insufficient rGO present to hold SiOC particles together and maintain electronic conducting network in the electrode. SEM images (b) top view and (c) cross-section view before cycling. (d) Digital picture and (e) SEM image of cycled electrode, which was dissembled in the lithiated state after 33 cycles. Arrows in the image indicate micro-cracks most likely due to cycling at high current density. Scale bar is 20 μm.

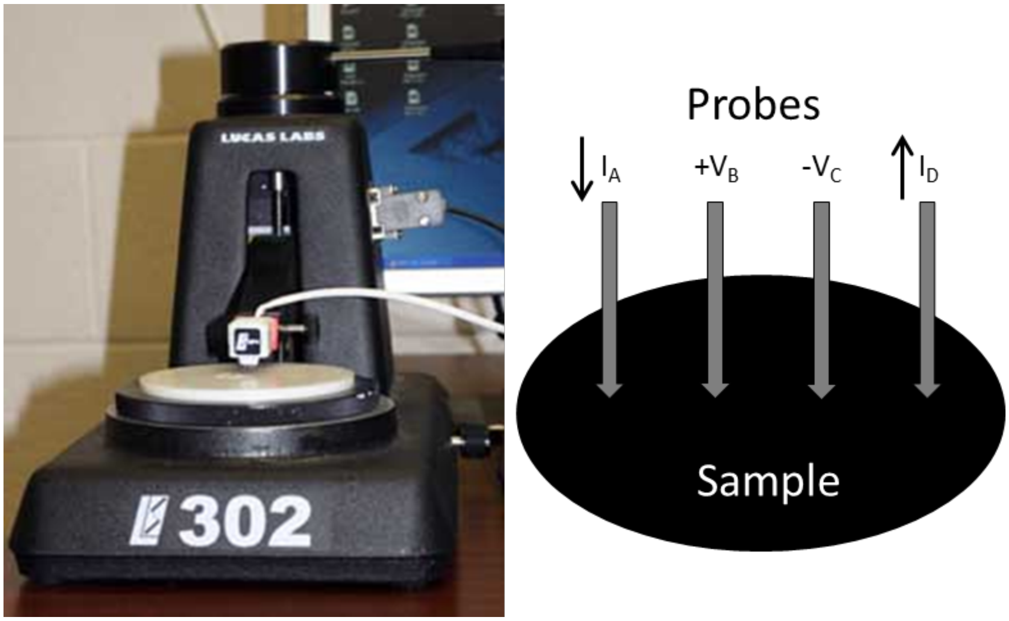


**SUPPLEMENTARY FIGURE 16:** Experimental setup for the four-point resistivity measurements.

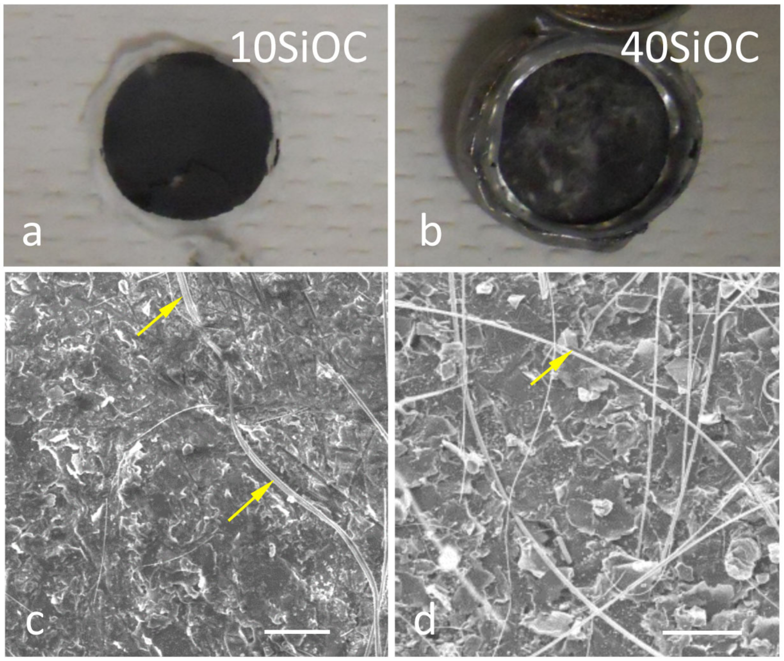


**SUPPLEMENTARY FIGURE 17: Post-cycling analysis.** (a, b) Digital camera images of 10SiOC and 40SiOC cycled electrodes and (c, d) corresponding SEM images. Cells were opened in lithiated state after 1020 cycles. Scale bar is 40 µm.

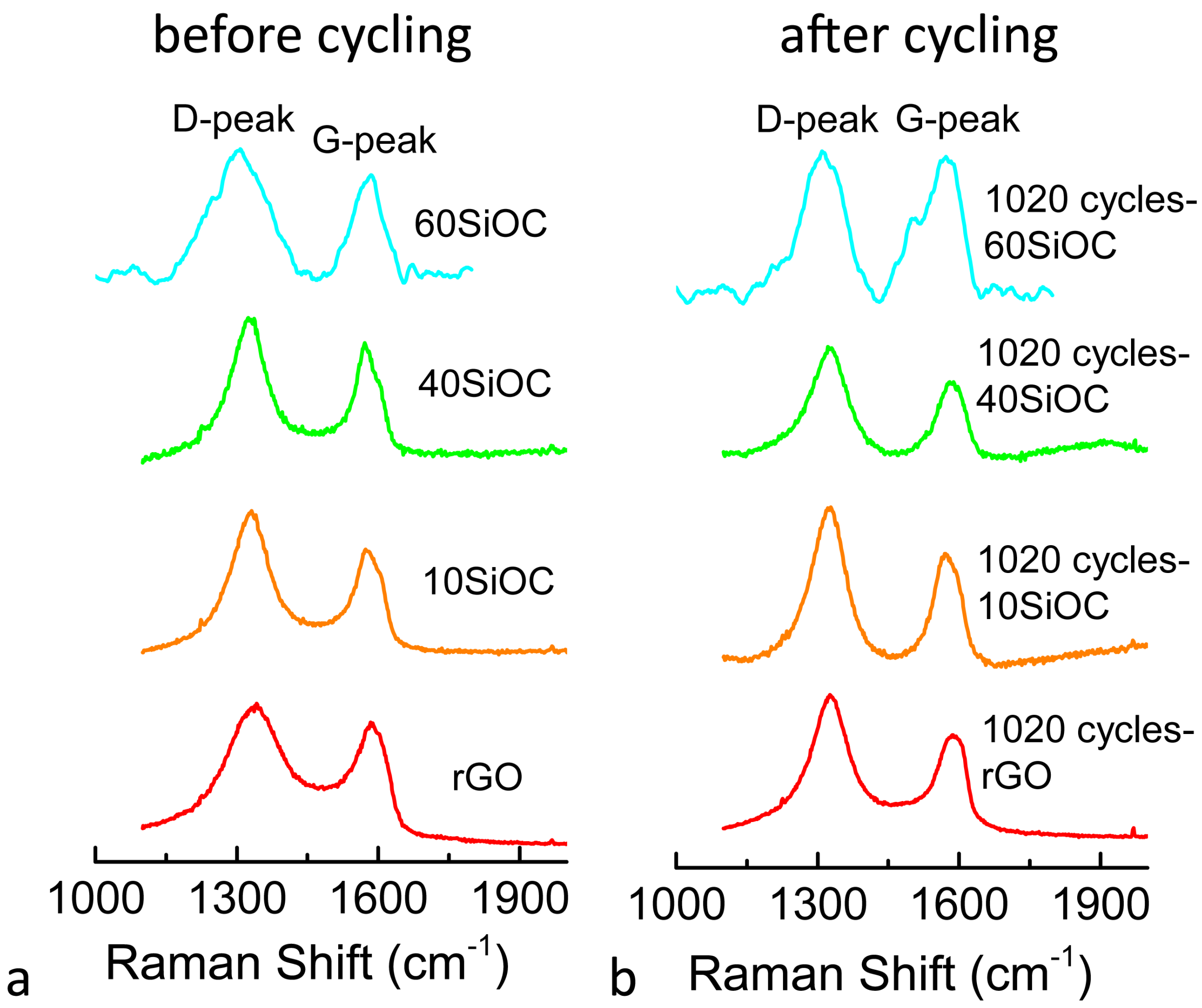


**SUPPLEMENTARY FIGURE 18: Raman analysis of various electrodes.** (a) before and (b) after the electrochemical testing. The electrodes were largely intact even upon cycling for 1020 cycles.

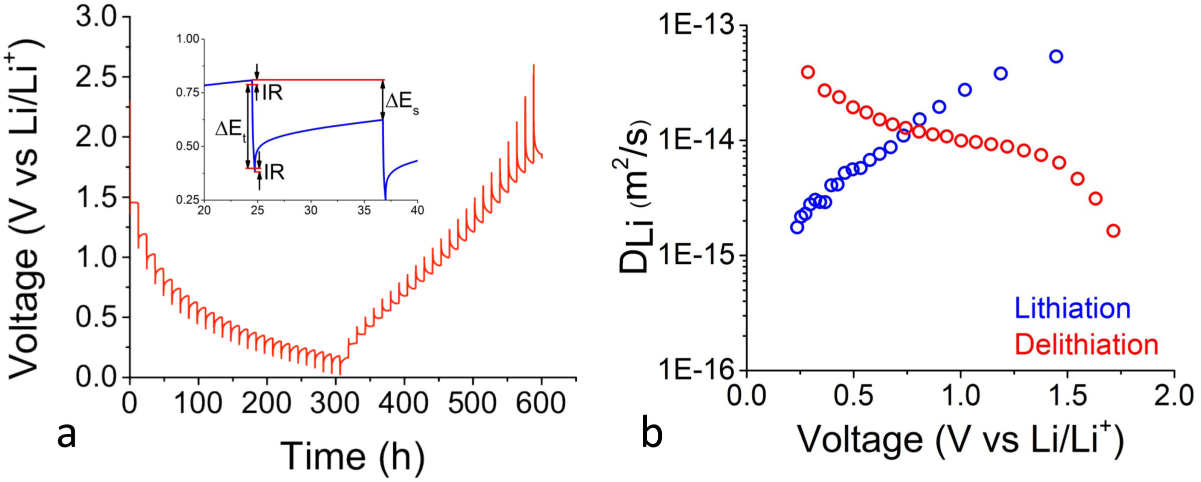


**SUPPLEMENTARY FIGURE 19: Room temperature (~25 °C) GITT Data.** (a) Charge and discharge cycles with 15 min of current pulse at 100 mA $g^{-1}_{electrode}$ followed by 12 h of relaxation for 60SiOC. (b) Calculated diffusion coefficient based on the relaxation steps at the corresponding voltages.

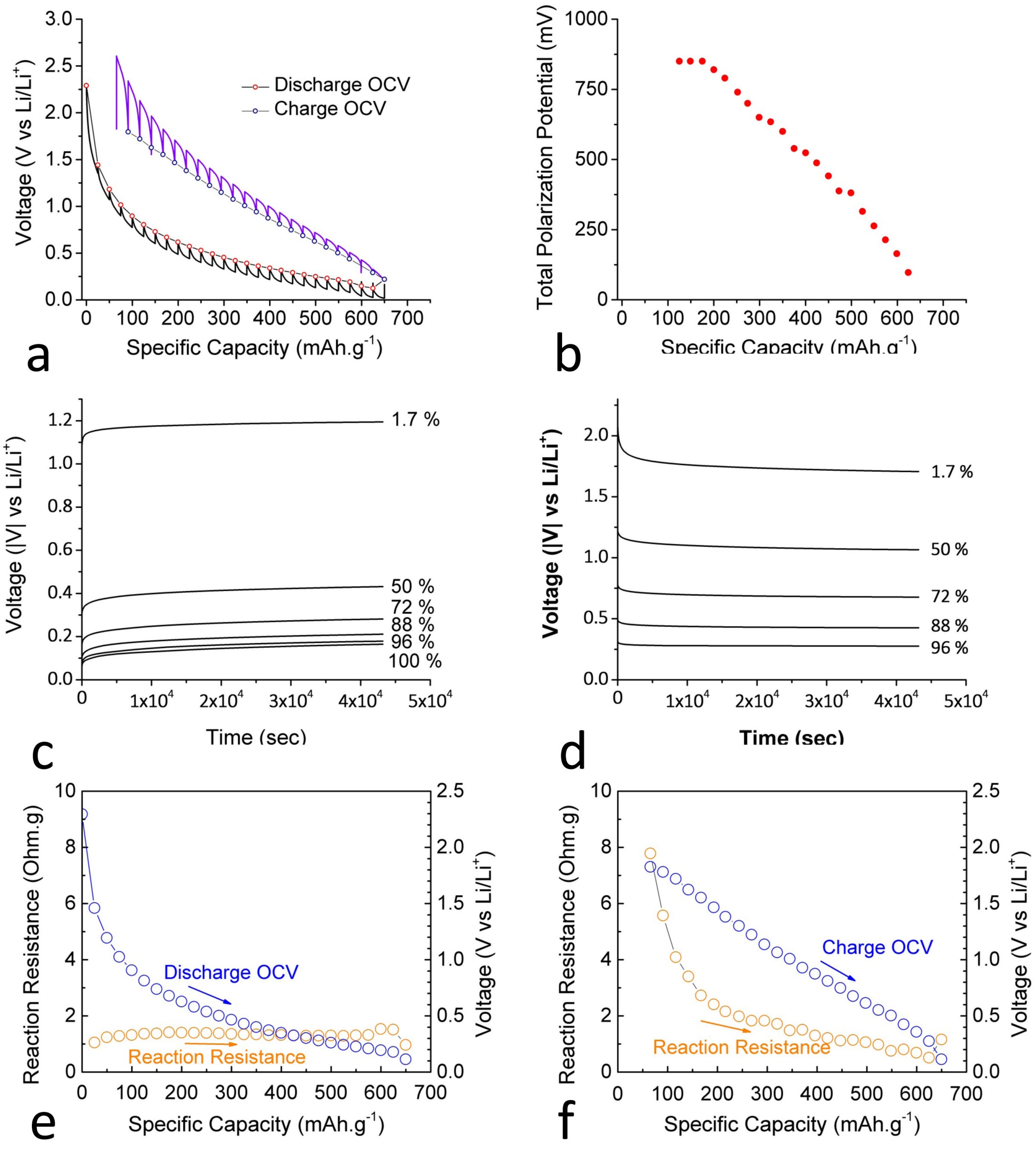


**SUPPLEMENTARY FIGURE 20: Additional electrochemical data for 60SiOC electrode at ~25 °C.** (a) Charge-discharge curve derived from titration study. (b) Polarization potential at different states of charge. Time-dependent change in the open-circuit voltage at different SOC during (c) insertion and (d) extraction half cycles (% of total electrode capacity). Calculated reaction resistance to Li-insertion (e) and extraction (f).

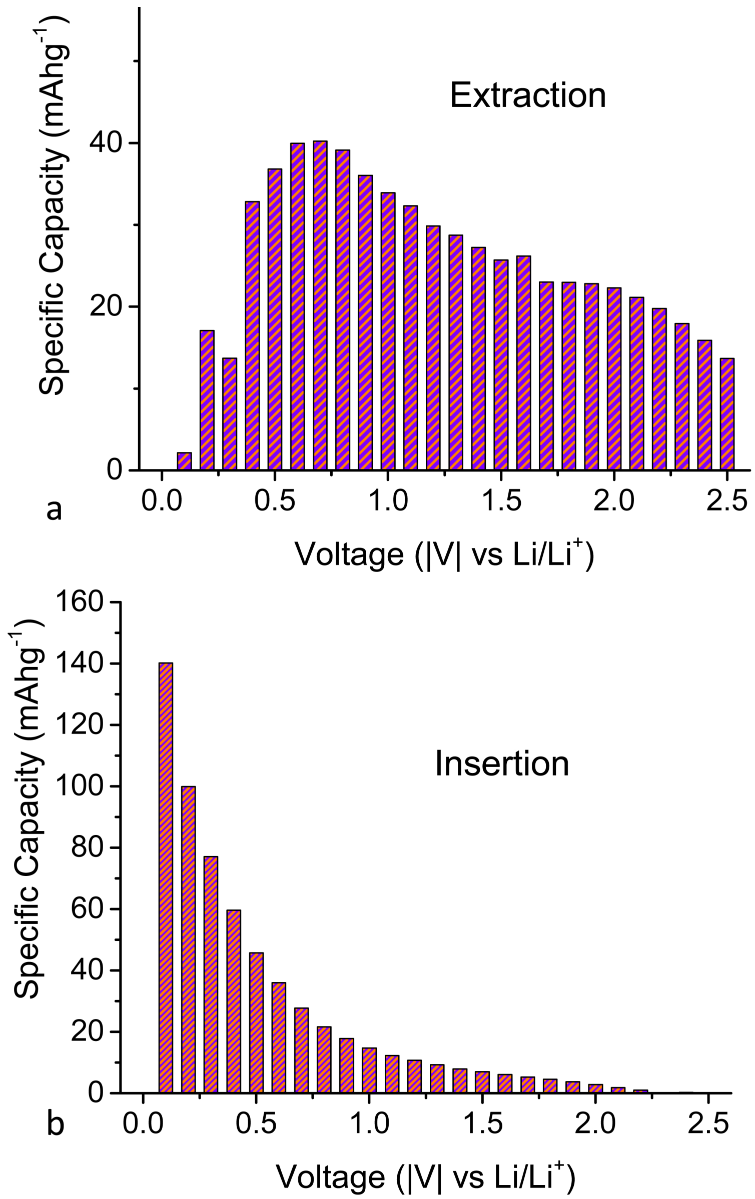


**SUPPLEMENTARY FIGURE 21: The Density of State (DOS) for 60SiOC electrode at ~25 °C.** (a) lithium extraction and (b) insertion. Extraction shows a much wide voltage distribution. Li extraction sees a shift of ~0.5 V (almost same as the voltage hysteresis).

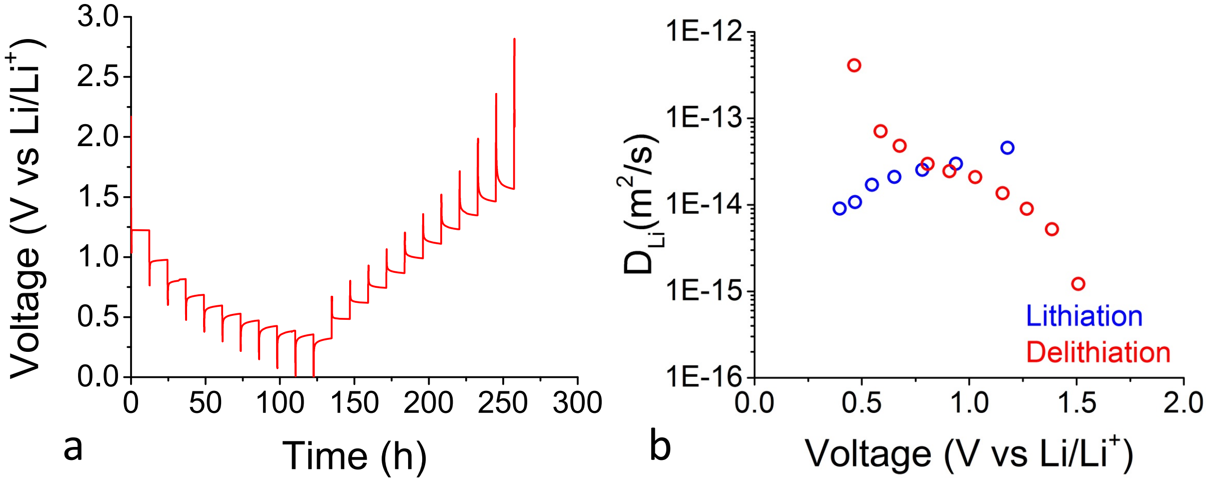


**SUPPLEMENTARY FIGURE 22: Low temperature (~-15 °C) GITT Data for 60SiOC electrode.** (a) Charge and discharge cycles with 15 min of current pulse at 100 mA $g^{-1}_{electrode}$ followed by 12 h of relaxation. (b) Calculated diffusion coefficient based on the relaxation steps at the corresponding voltages.

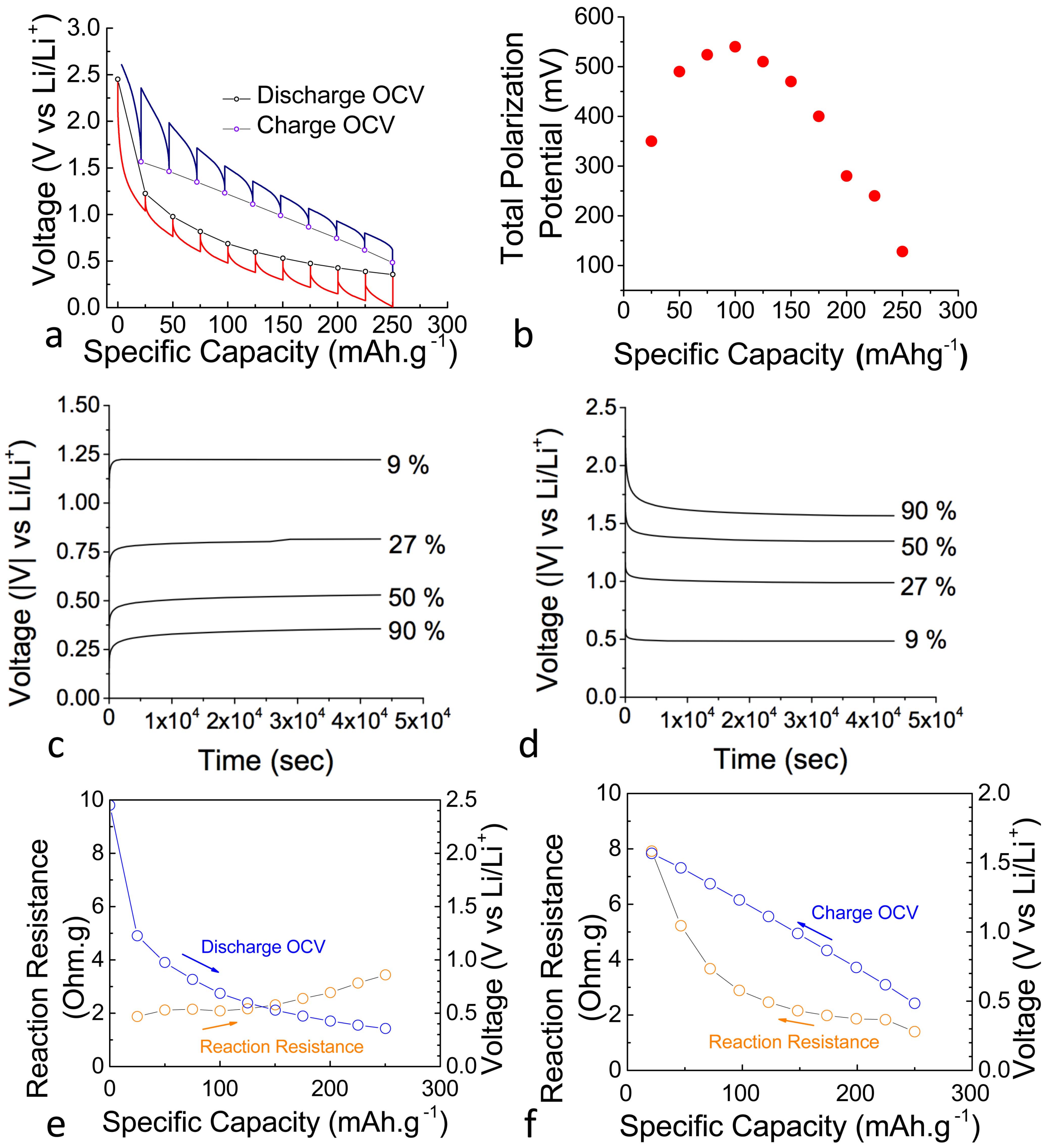


**SUPPLEMENTARY FIGURE 23: Additional electrochemical data for 60SiOC electrode at ~-15 °C.** (a) Charge-discharge curve derived from titration study. (b) Total polarization potential at different SOC. Time dependent change in the open-circuit voltage at different SOC during (c) insertion and (d) extraction halves (% of total electrode capacity). Calculated reaction resistance to Li-insertion (e) and extraction (f).

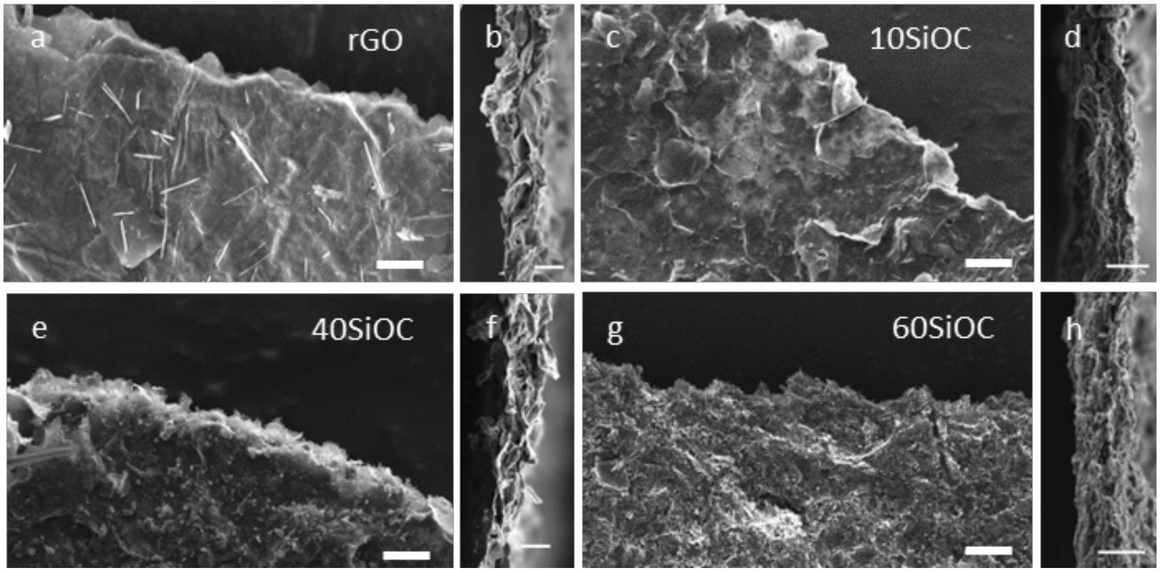


**SUPPLEMENTARY FIGURE 24: SEM after mechanical testing.** (a, c, e, g) Top and (b, d, f, h) cross-sectional SEM images of rGO, 10SiOC, 40SiOC and 60SiOC specimens. The scale bar is 20 μm.

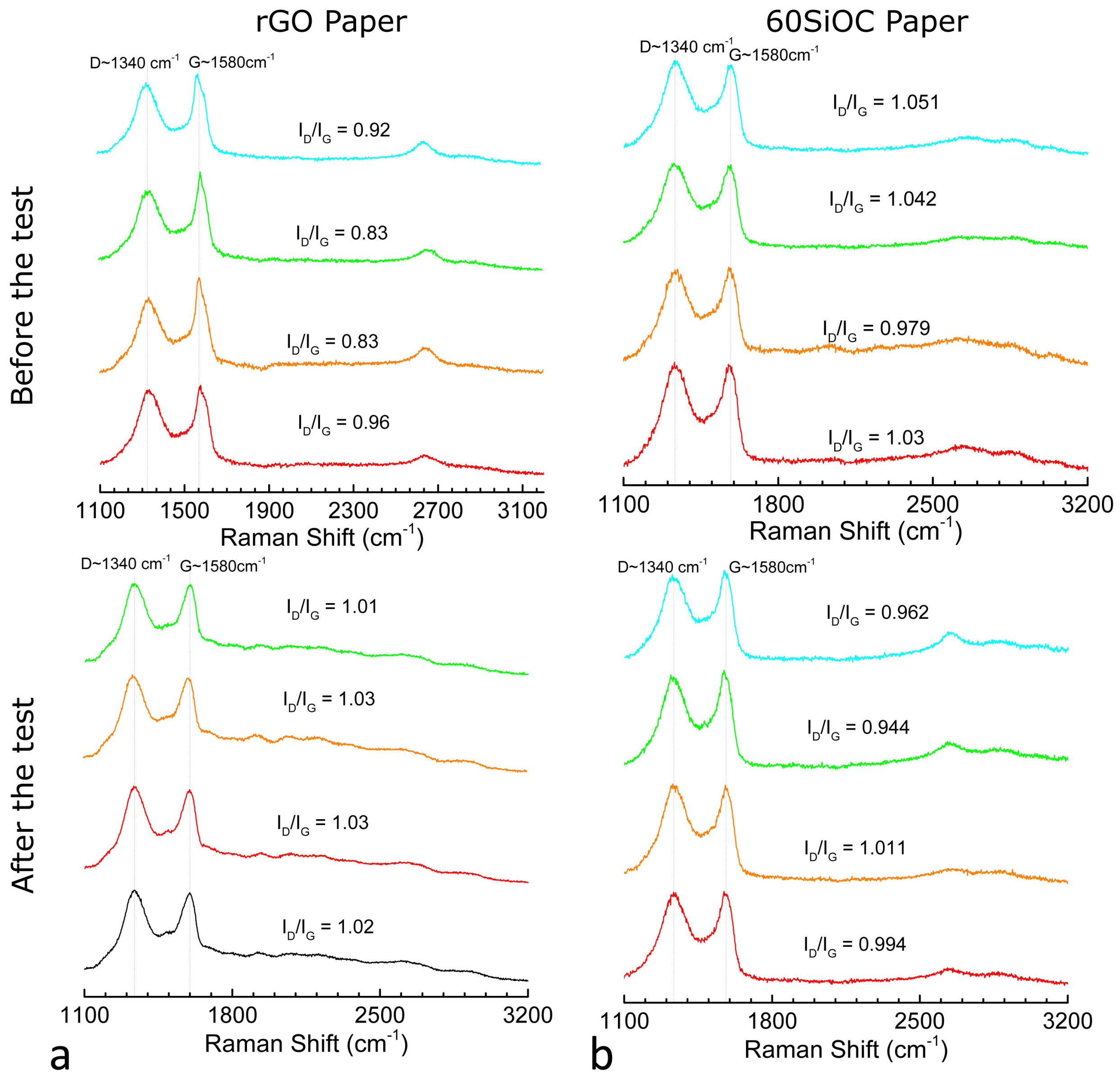


**SUPPLEMENTARY FIGURE 25: Raman data before and after mechanical testing.** Raman spectrum ($I_d/I_g$) of the composite papers (a) pre and (b) post tensile testing. Each plot is an average of four data points with accumulation time of 20 seconds each.

## Supplementary Tables

**SUPPLEMENTARY TABLE 1:** Composition measured by SEM, XPS, and Combustion-Fusion-IR-Thermal conductivity techniques.

| Sample ID | Atomic Concentrations (%) | | | | Weight Concentrations (%) | | | |
|---|---|---|---|---|---|---|---|---|
| | C | O | Si | H | C | O | Si | H |
| Cross-linked Precursor (XPS) | 54.44 | 28.59 | 16.97 | - | 41.18 | 28.79 | 30.01 | - |
| SiOC (SEM EDS, surface) | 37 | 46 | 17 | - | 27 | 44 | 29 | - |
| SiOC (XPS, surface t=0 min) | 62.55 | 25.73 | 11.72 | - | 50.35 | 27.57 | 22.06 | - |
| SiOC (XPS, sputter t=20 min) | 47.94 | 22.87 | 29.2 | - | 32.68 | 20.76 | 46.55 | - |
| SiOC (XPS, sputter t=40 min) | 49.12 | 20.72 | 30.16 | - | 33.36 | 18.73 | 47.89 | - |
| SiOC (XPS, sputter t=60 min) | 49.86 | 19.46 | 30.68 | - | 33.8 | 17.56 | 48.63 | - |
| SiOC (XPS, sputter t=80 min) | 50.78 | 18.44 | 30.78 | - | 34.47 | 16.66 | 48.85 | - |
| SiOC (Bulk powder) | 51.24 | 19.79 | 23.85 | 5.09 | 38.3 | 19.7 | 41.68 | 0.31 |
| *All XPS atomic percentage measurements are accurate up to approximately 15%.* | | | | | | | | |

**SUPPLEMENTARY TABLE 2:** Summary of TGA data for composite papers prior to thermal reduction (corresponding to Fig. 2 main text).

| Sample | Weight Loss at Oxidation Temperature Range (%) | | | |
|---|---|---|---|---|
| | (50 to 400) °C ±1 s.d. | | (400 to 800)°C ±1 s.d. | |
| GO | 52.0 | 0.2 | 44.4 | 0.5 |
| 10SiOC | 29.1 | 0.0 | 44.8 | 0.1 |
| 40SiOC | 16.6 | 0.3 | 33.0 | 0.8 |
| 60SiOC | 11.3 | 0.3 | 23.6 | 0.9 |
| 80SiOC | 3.8 | 0.2 | 15.9 | 1.1 |
| SiOC | - | - | 4.8 | 0.6 |

**SUPPLEMENTARY TABLE 3**: 60SiOC paper electrode testing results. Current density for extraction is 100 mA $g_{electrode}^{-1}$.

| Cycle Number | Discharge Capacity (mAh $g_{electrode}^{-1}$) | Charge Capacity (mAh $g_{electrode}^{-1}$) | Efficiency (%) | Charge Current Density (mA $g_{electrode}^{-1}$) |
|---|---|---|---|---|
| 1 | 1032.2241 | 702.1838 | 68.0263 | 100 |
| 2 | 690.7436 | 667.9689 | 96.7029 | |
| 3 | 666.7953 | 650.5418 | 97.5624 | |
| 4 | 653.3472 | 641.4820 | 98.1839 | |
| 5 | 640.2787 | 629.8547 | 98.3720 | |
| 6 | 632.6639 | 611.4274 | 96.6433 | 200 |
| 7 | 611.7468 | 604.1980 | 98.7660 | |
| 8 | 608.4371 | 602.1728 | 98.9704 | |
| 9 | 603.7679 | 598.4485 | 99.1190 | |
| 10 | 596.6603 | 591.4099 | 99.1200 | |
| 11 | 598.8522 | 579.5187 | 96.7716 | 400 |
| 12 | 584.8954 | 580.1059 | 99.1811 | |
| 13 | 578.4616 | 573.1897 | 99.0886 | |
| 14 | 577.076 | 572.2012 | 99.1553 | |
| 15 | 574.589 | 571.1472 | 99.4010 | |
| 16 | 574.2987 | 549.9743 | 95.7645 | 800 |
| 17 | 560.1708 | 553.2306 | 98.7611 | |
| 18 | 558.6627 | 550.7485 | 98.5834 | |
| 19 | 561.6413 | 576.3575 | 102.6202 | |
| 20 | 460.1736 | 559.9298 | 121.6779 | |
| 21 | 571.8498 | 546.4572 | 95.5596 | 1600 |
| 22 | 548.8859 | 542.8219 | 98.8952 | |
| 23 | 550.3691 | 545.7319 | 99.1574 | |
| 24 | 553.3016 | 548.4487 | 99.1229 | |
| 25 | 555.8721 | 551.0029 | 99.1240 | |
| 26 | 560.1028 | 684.1608 | 122.1491 | 2400 |
| 27 | 544.3323 | 543.3356 | 99.8169 | |
| 28 | 545.9675 | 541.4211 | 99.1673 | |
| 29 | 544.6043 | 542.4265 | 99.6001 | |
| 30 | 545.275 | 541.7531 | 99.3541 | |
| 31 | 545.4481 | 624.4166 | 114.4777 | 100 |
| 32 | 632.0522 | 629.7266 | 99.6320 | |
| 33 | 632.5852 | 628.3935 | 99.3374 | |
| 34 | 632.8094 | 628.9953 | 99.3973 | |
| 35 | 634.517 | 629.6484 | 99.2327 | |
| 36 | 633.497 | 629.7021 | 99.4010 | |
| 37 | 632.1245 | 626.6654 | 99.1364 | |
| 38 | 628.7229 | 625.3246 | 99.4595 | |
| 39 | 630.888 | 625.9345 | 99.2148 | |
| 40 | 628.8819 | 626.2669 | 99.5842 | |

**SUPPLEMENTARY TABLE 4**: Summary of experimental results of various freestanding graphene hybrid papers

| Freestanding Electrode Material | 1st cycle charge capacity, mAh $g^{-1}$ | 1st cycle efficiency, % | Charge current density, mA $g^{-1}$ | Charge capacity (cycle number), mAh $g^{-1}$ | Efficiency, % | Ref. no. |
|---|---|---|---|---|---|---|
| 60SiOC | 702 | 68 | 100 | 588(1020) | 99.6 | **This work** |
| | | | 2400 | 543 | ~100 | |
| SiOC (bulk/traditional anode)* | 958 | 77 | 100 | 700(60) | ~100 | 1 |
| | | | 2400 | 50 | NA | |
| Graphite (bulk/traditional)* | 298 | NA | 50 | 240(50) | NA | 2 |
| r-Graphene oxide | 680 | 12.4 | 50 | 84(2) | 80 | |
| Graphene-800 °C | 1050 | 47 | 50 | 301(10) | NA | |
| rGO-Ar-III (Holey graphene) | 403 | 49 | 50 | 403(10) | 99.8 | 3 |
| | | | 2000 | 178 | 99.8 | |
| | | | 10000 | ~75 (1000) | 99.8 | |
| Si/Graphene | ~900 | ~41 | 50 | 708(100) | NA | 4 |
| SiNW/Graphene | ~590 | ~51 | 50 | ~500(20) | NA | 5 |
| GeNW/Graphene | ~860 | ~81 | 65.4 | ~610(20) | NA | |
| SiNW/Graphene/rGO | ~2000 | ~45 | 840 | 1650(50) | 98 | 6 |
| $TiO_2$/Graphene | 237 | 58.5 | 200 | ~157(100) | 99 | 7 |
| | | | 2000 | ~122(100) | 95 | |
| | | | 4000 | ~100 | NA | |
| $MnO_2$/Graphene | ~686 | ~54 | 100 | ~500(70) | NA | 8 |
| | | | 1600 | ~200 | NA | |
| $SnO_2$/Graphene | ~700 | NA | 100 | 438(50) | NA | 9 |
| $SnO_2$/Graphene/CNT | ~830 | ~52 | 250 | 635(80) | NA | 10 |
| | | | 1000 | 400(50) | NA | |
| $SnO_2$/Graphene | ~602 | ~48 | 250 | 600(10) | NA | |
| | | | 1000 | 390 | NA | |
| $Fe_3O_4$/Carbon/CNT | 821 | 71.5 | 156 | ~900(10) | NA | 11 |
| MWCNT | 206 | 34 | 100 | 190(30) | | 12 |
| Si/MWCNT | 1058 | 79.6 | 100 | 942(30) | NA | |
| SWCNT | ~263 | ~18 | 25 | NA | NA | 13 |
| $SnO_2$/SWCNT | ~570 | ~31 | 25 | NA | NA | |

**(Bulk material i.e., not freestanding with anode prepared on copper current collector)*.

**SUPPLEMENTARY TABLE 5:** Summary of the electrical conductivity test data.

| Material | Resistance, Ω | Resistivity, Ω cm | Conductivity, S $cm^{-1}$ |
|---|---|---|---|
| rGO | 40 | 0.04 | 27.59 |
| 10SiOC | 210 | 0.95 | 1.05 |
| 40SiOC | 480 | 4.35 | 0.23 |
| 60SiOC | 580 | 18.39 | 0.05 |
| 80SiOC | 1500 | 47.57 | 0.02 |

**SUPPLEMENTARY TABLE 6:** Summary of the Raman data for various electrodes pre and post-cycling.

| Sample | Pre-cycling | | | Post-cycling | | |
|---|---|---|---|---|---|---|
| | $I_d$ | $I_g$ | $I_d / I_g$ | $I_d$ | $I_g$ | $I_d / I_g$ |
| 60SiOC | 1329.96 | 1571.84 | 0.85 | 1323.15 | 1583.77 | 0.84 |
| 40SiOC | 1329.96 | 1576.95 | 0.84 | 1331.66 | 1597.39 | 0.83 |
| 10SiOC | 1331.66 | 1576.95 | 0.84 | 1326.55 | 1575.25 | 0.84 |
| rGO | 1341.88 | 1588.88 | 0.84 | 1326.55 | 1590.58 | 0.83 |

## Supplementary Notes

### Supplementary Note 1: Electrical Conductivity Data

Van der Pauw's four-point resistivity measurement technique[14] is a preferable as it helps eliminate spreading resistances and contact resistance that can be associated with the two-point probe measurements. In order to find the conductivity of a material, four contacts must be applied to each edge of the surface, equal distance apart. Apply labels to each of the four points, A, B, C and D. Current is applied to A and D contacts and voltage to B and C contacts. The resistance of the material can be determined by

$$R_{AD,BC} = \frac{(V_B - V_C)}{I_{AD}} \quad \Omega$$

With the other orientation, where current is applied to contacts B and D and voltage applied to A and C, the resistance can be found by

$$R_{BD,AC} = \frac{(V_A - V_C)}{I_{BD}} \quad \Omega$$

Once the two resistance measurements are complete, the resistivity can be calculated by

$$R_{AD,BC} = \frac{\pi t}{\mathbf{ln(2)}} * \frac{(R_{AD,BC} + R_{BD,AC})f}{\mathbf{2}} \quad \Omega\ cm$$

where 't' is the sample thickness and f(=1) is a correction factor between 0 and 1 to take into account the asymmetry in the sample. The conductivity can be found out by

$$C = \frac{\mathbf{1}}{\rho} \quad S\ cm^{-1}$$

## Supplementary Note 2: Electrochemical Data for Cell Tested at Low Temperature (~-15 °C)

Electrochemical testing at low temperature was carried out on the best performing specimen i.e., 60SiOC. The cell was placed in an airtight container while being cycled at a constant current rate of 100 mA $g^{-1}_{electrode}$ in the cooling unit (refrigerator). After cycling for 10 cycles at room temperature (~25 °C), the cell was rested in the cooling unit (freezer of a household refrigerator) for 1 day to uniformly cool the cell to ~-15 °C before cycling it at low temperature for 50 cycles. The cell was rested at room temperature after $60^{th}$ cycle for 1 day before cycling it at room temperature for another 10 cycles.

## Supplementary Note 3: Electrochemical Titration Studies

In the galvanostatic intermittent titration technique (GITT) experiment, charge was inserted (or withdrawn) by applying a current pulse of 100 mAh $g^{-1}_{electrode}$ for 15 min, followed by 12 h of relaxation between pulses during which the change in potential with time was measured. Magnitude of voltage increased during the relaxation period in the insertion half but it decreased in the extraction half of the cycle.[15, 16] The solid state lithium ion diffusion coefficients were determined using

$$D_{GITT} \approx \frac{4}{\pi\tau}\left(\frac{m_B V_M}{M_B S}\right)^2\left(\frac{\Delta E_S}{\Delta E_T}\right)^2 \quad m^2 s^{-1}$$

Where '$\tau$' is pulse time (15 min), '$m_B$' is electrode mass (3.23 mg), '$V_M$' is molar volume (5.3 cc $mol^{-1}$), '$M_B$' molecular weight (12 g $mol^{-1}$) and 'S' is surface area (1.6 $cm^2$), '$\Delta E_S$' and '$\Delta E_T$' are voltage steps as shown in insert in Supplementary Fig. 19. The open circuit voltage (OCV) at the end of each

relaxation is considered as the thermodynamic equilibrium potential. For this study only the best performing electrode i.e., 60SiOC was used.

Total polarization potential was then derived from the charge/discharge curve of the titration experiment. In the charge/discharge curve that was obtained from the GITT experiment, the end point of each relaxation period was joined together to form a curve that is shown as a dotted line in Supplementary Fig. 20a. The difference in voltage at each point in the curve is the total polarization potential, which is shown in Supplementary Fig. 20b. Supplementary Fig. 20c,d shows the change in voltage during the relaxation period at different states of charge (SOC). The SOC is defined as the percentage of total capacity present in the electrode at that specific voltage. Further, the reaction resistance for the electrode was calculated for both the discharge and charge half of the GITT experiment by simply calculating the ratio of overpotential to the current density at each pulse (Supplementary Fig. 20e,f).

Later to determine the distribution of density of states of the electrode material, voltage at specific decrement is applied till charges occupy the levels above that voltage (or the applied current becomes zero). The total charge that is inserted into the electrode (equal to the integral value of the current) at that voltage gives the density of charge that can be stored at that level. In the next step charge is stored at the next energy level and so on. In this way the density of charge stored at each voltage is determined. To determine the density of states during the extraction half cycle, voltage at specific increment is applied after full insertion of lithium in the electrode.

The cell was later subjected to testing at low temperature (~-15 °C) in a similar manner.

## Supplementary References


1. Ahn, D. & Raj, R. Cyclic stability and C-rate performance of amorphous silicon and carbon based anodes for electrochemical storage of lithium. *J. Power Sources* **196**, 2179-2186 (2011).

2. Wang, C., Li, D., Too, C. O. & Wallace, G. G. Electrochemical properties of graphene paper electrodes used in lithium batteries. *Chem. Mater.* **21**, 2604-2606 (2009).

3. Zhao, X., Hayner, C. M., Kung, M. C. & Kung, H. H. Flexible holey graphene paper electrodes with enhanced rate capability for energy storage applications. *ACS Nano* **5**, 8739-8749 (2011).

4. Wang, J. Z., Zhong, C., Chou, S. L. & Liu, H. K. Flexible free-standing graphene-silicon composite film for lithium-ion batteries. *Electrochem. Commun.* **12**, 1467-1470 (2010).

5. Chockla, A. M. *et al.* Electrochemical lithiation of graphene-supported silicon and germanium for rechargeable batteries. *J. Phys. Chem. C* **116**, 11917-11923 (2012).

6. Wang, B. *et al.* Adaptable silicon-carbon nanocables sandwiched between reduced graphene oxide sheets as lithium ion battery anodes. *ACS Nano* **7**, 1437-1445 (2013).

7. Hu, T. *et al.* Flexible free-standing graphene-$TiO_2$ hybrid paper for use as lithium ion battery anode materials. *Carbon* **51**, 322-326 (2013).

8. Yu, A. *et al.* Free-standing layer-by-layer hybrid thin film of graphene-$MnO_2$ nanotube as anode for lithium ion batteries. *J. Phys. Chem. Lett.* **2**, 1855-1860 (2011).

9. Liang, J., Zhao, Y., Guo, L. & Li, L. Flexible free-standing graphene/$SnO_2$ nanocomposites paper for Li-ion battery. *ACS Appl. Mater. Interfaces* **4**, 5742-5748 (2012).

10. Zhang, B., Zheng, Q. B., Huang, Z. D., Oh, S. W. & Kim, J. K. $SnO_2$-graphene-carbon nanotube mixture for anode material with improved rate capacities. *Carbon* **49**, 4524-4534 (2011).

11. Jia, X. *et al.* Building robust architectures of carbon and metal oxide nanocrystals toward high-performance anodes for lithium-ion batteries. *ACS Nano* **6**, 9911-9919 (2012).

12. Yue, L., Zhong, H. & Zhang, L. Enhanced reversible lithium storage in a nano-Si/MWCNT free-standing paper electrode prepared by a simple filtration and post sintering process. *Electrochim. Acta* **76**, 326-332, (2012).

13. Noerochim, L., Wang, J. Z., Chou, S. L., Wexler, D. & Liu, H. K. Free-standing single-walled carbon nanotube/$SnO_2$ anode paper for flexible lithium-ion batteries. *Carbon* **50**, 1289-1297 (2012).

14. Van der Pauw, L.J., A method of measuring specific resistivity and hall effect of discs of arbitrary shape. *Philips Research Reports*, **13**, 1, 1-9 (1958).

15. Weppner, W. & Huggins, R. A. Determination of the kinetic parameters of mixed-conducting electrodes and application to the system $Li_3Sb$. *J. Electrochem. Soc.* **124**, 1569-1578 (1977).

16. Wen, C. J., Boukamp, B. A., Huggins, R. A. & Weppner, W. Thermodynamic and mass transport properties of "LiAl". *J. Electrochem. Soc.* **126**, 2258-2266 (1979).
